# Daily Forecasting of Regional Epidemics of Coronavirus Disease with Bayesian Uncertainty Quantification, United States

**DOI:** 10.3201/eid2703.203364

**Published:** 2021-03

**Authors:** Yen Ting Lin, Jacob Neumann, Ely F. Miller, Richard G. Posner, Abhishek Mallela, Cosmin Safta, Jaideep Ray, Gautam Thakur, Supriya Chinthavali, William S. Hlavacek

**Affiliations:** Los Alamos National Laboratory, Los Alamos, New Mexico, USA (Y.T. Lin, W.S. Hlavacek);; Northern Arizona University, Flagstaff, Arizona, USA (J. Neumann, E.F. Miller, R.G. Posner);; University of California, Davis, California, USA (A. Mallela);; Sandia National Laboratories, Livermore, California, USA (C. Safta, J. Ray);; Oak Ridge National Laboratory, Oak Ridge, Tennessee, USA (G. Thakur, S. Chinthavali)

**Keywords:** mathematical model, statistics, uncertainty, epidemics, coronavirus disease, COVID-19, severe acute respiratory syndrome coronavirus 2, SARS-CoV-2, zoonoses, viruses, United States, Bayesian statistics, compartmental model

## Abstract

To increase situational awareness and support evidence-based policymaking, we formulated a mathematical model for coronavirus disease transmission within a regional population. This compartmental model accounts for quarantine, self-isolation, social distancing, a nonexponentially distributed incubation period, asymptomatic persons, and mild and severe forms of symptomatic disease. We used Bayesian inference to calibrate region-specific models for consistency with daily reports of confirmed cases in the 15 most populous metropolitan statistical areas in the United States. We also quantified uncertainty in parameter estimates and forecasts. This online learning approach enables early identification of new trends despite considerable variability in case reporting.

Coronavirus disease (COVID-19), caused by severe acute respiratory syndrome coronavirus 2 (SARS-CoV-2) (*1*), was detected in the United States in January 2020 (*2*). Researchers documented deaths in the United States caused by COVID-19 in February (*3*). Thereafter, surveillance testing expanded nationwide (*4*). These and other efforts revealed community spread across the United States and exponential growth of new COVID-19 cases throughout most of March. Growth of cases during February–April had a doubling time of 2–3 days (*5*), similar to the doubling time of the initial outbreak in China (*6*). The rapid increase in cases prompted broad adoption of social distancing practices such as teleworking, travel restrictions, use of face masks, and government mandates prohibiting public gatherings (*7*). The United States soon became a hotspot of the COVID-19 pandemic. In the United States, detection of new cases peaked in late April and steadily declined until mid-June (*4*). The decline in case numbers suggest that mandates and social distancing interventions effectively slowed COVID-19 transmission. Efforts to quantify the effects of these measures indicate that they substantially reduced disease prevalence (*8*,*9*).

In mid-June and mid-September 2020, the daily incidence of COVID-19 cases in the United States increased a second and third time (*4*). Public health officials must effectively monitor ongoing COVID-19 transmission to quickly respond to dangerous upticks in disease. To contribute to situational awareness of COVID-19 transmission dynamics, we developed a mathematical model for the daily incidence of COVID-19 in each of the 15 most populous US metropolitan statistical areas (MSAs) (*10*). Each model is composed of ordinary differential equations (ODEs) characterizing the dynamics of various populations, including subpopulations that did or did not practice social distancing.

We used online learning to calibrate our models for consistency with historical case reports. We also applied Bayesian methods to quantify uncertainties in predicted detection of new cases. This approach enabled identification of new epidemic trends despite variability in case detection. These findings can inform policymakers designing evidence-based responses to regional COVID-19 epidemics in the United States.

## Methods

### Data Used in Online Learning

We obtained reports of new confirmed cases from the GitHub repository maintained by The New York Times newspaper (*11*). Each day, at varying times of day, we updated the model using cumulative data since January 21, 2020. The data in this analysis is from January 21–June 26, 2020. We aggregated county-level data to obtain case counts for each of the 15 most populous US MSAs, which encompass the following cities: New York City, New York; Los Angeles, California; Chicago, Illinois; Dallas, Texas; Houston, Texas; Washington, DC; Miami, Florida; Philadelphia, Pennsylvania; Atlanta, Georgia; Phoenix, Arizona; Boston, Massachusetts; San Francisco, California; Riverside, California; Detroit, Michigan; and Seattle, Washington. 

The political entities comprising each MSA are those delineated by the federal government (*10*). The number of political units (i.e., counties and independent cities) in the MSAs of interest ranged from 2 (for the Los Angeles and Riverside MSAs) to 29 (for the Atlanta MSA). The median number of counties in an MSA was 7; the mean was 10. The number of states encompassing an MSA ranged from 1 (for 8/15 MSAs) to 4 (for Philadelphia). The median number of encompassing states was 1; the mean was 2.

### COVID-19 Transmission Model and Parameters

We used daily reports of new cases to parameterize a compartmental model for the regional COVID-19 epidemic in each of the 15 MSAs of interest. Until June 2020, we also parameterized curve-fitting models. However, curve-fitting models can generate only single-peak epidemic curves, so we abandoned this approach after the MSAs of interest all experienced multiple waves of disease (Appendix 1).

**Figure 1:**
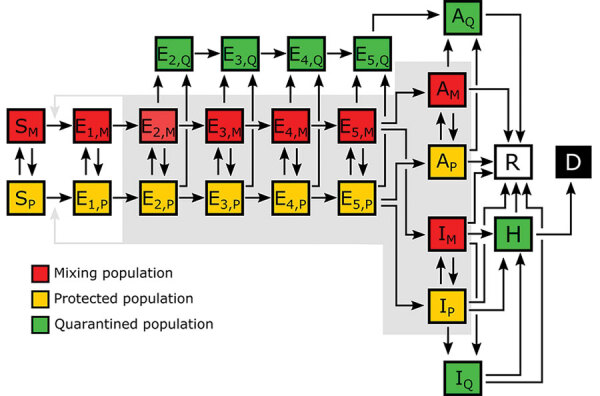
Illustration of the populations and processes considered in a mechanistic compartmental model of coronavirus disease daily incidence during regional epidemics, United States, 2020. The model accounts for susceptible persons (*S*), exposed persons without symptoms in the incubation phase of disease (*E*), asymptomatic persons in the immune clearance phase of disease (*A*), mildly ill symptomatic persons (*I*), severely ill persons in hospital or at home (*H*), recovered persons (*R*), and deceased persons (*D*). The model also accounts for social distancing, which establishes mixing (_M_) and protected (_P_) subpopulations; quarantine driven by testing and contact tracing, which establishes quarantined subpopulations (_Q_); and self-isolation spurred by symptom awareness. Persons who are self-isolating because of symptoms are considered to be members of the *I_Q_* population. The incubation period is divided into 5 stages (*E_1_*–*E_5_*), which enables the model to reproduce an empirically determined (nonexponential) Erlang distribution of waiting times for the onset of symptoms after infection (*12*). The exposed population consists of persons incubating virus and is comprised of presymptomatic and asymptomatic persons. The *A* populations consist of asymptomatic persons in the immune clearance phase. The gray background indicates the populations that contribute to disease transmission. An auxiliary measurement model (Appendix Equations 23, 24) accounts for imperfect detection and reporting of new cases. Only symptomatic cases are assumed to be detectable in surveillance testing. Red indicates the mixing population; yellow indicates the protected population; green indicates the quarantined population; white indicates the recovered population; black indicates the deceased population.

Each MSA-specific model accounted for 25 populations (Figure 1; Appendix 1 Figure 1). We considered infectious persons to be exposed and incubating virus (i.e., presymptomatic), asymptomatic while clearing virus, or symptomatic. The parameter *ρ_E_* characterized the relative infectiousness of exposed persons and *ρ_A_* characterized that of asymptomatic persons compared with symptomatic persons. In our model, infected persons quarantined with rate constant *k_Q_* and symptomatic persons with mild disease quarantined with rate constant *j_Q_*. We modeled social distancing by enabling the movement of susceptible and infectious persons between mixing and socially distanced (i.e., protected) populations. The size of the protected population was determined by 2 parameters: *λ_i_*, a rate constant; and *p_i_*, a steady-state population setpoint, where index *i* refers to the current social distancing period. The model accounts for varying adherence to social distancing practices over time by using *n* distinct social distancing periods after an initial period of social distancing. Persons in the protected population were less likely to be infected and less likely to transmit disease by a factor *m_b_*. Within the mixing population, disease was transmitted with rate constant *β*. The model reproduced a nonexponentially distributed incubation period by dividing the incubation period into 5 sequential stages of equal mean duration, given by 1/*k_L_*. We considered infected persons in the first stage of the incubation period to be noninfectious and undetectable. A fraction of exposed persons, *f_A_*, left the incubation period without symptoms. The remaining persons left with symptoms. The other symptomatic persons, *f_H_*, progressed to severe disease; the remainder had mild disease and recovered. The fraction of persons with severe disease who recovered is denoted as *f_R_*; the others died. We considered hospitalized persons (or those at home with severe disease) to be quarantined. Persons left the asymptomatic state with rate constant *c_A_*, left the mild disease state with rate constant *c_I_*, and left the severe disease/hospitalized state with rate constant *c_H_*.

The model consisted of 25 ODEs (Appendix 1 Equations 1–17). Each state variable of the model represented the size of a population. In addition to the 25 ODEs, we considered an auxiliary 1-parameter measurement model that related state variables to expected case reporting (Appendix 1 Equations 23, 24) and a negative binomial model for variability in new case detection (Appendix 1 Equations 25–27). We designed the model to consider multiple periods of social distancing with distinct setpoints for the quasistationary protected population size. The model always included an initial period of social distancing. The number of additional social distancing periods was given by *n*. Here, we considered only 2 cases: *n* = 0 and *n* = 1. We determined the best value of *n* by using model selection (Appendix 1).

The compartmental model and the auxiliary measurement model for *n* = 0 had a total of 20 parameters. We considered 6 of these parameters to have adjustable values (Table 1) and 14 to have fixed values (Tables 2, 3) (*12**–**20*; Appendix 1). The adjustable model parameters were *t_0_*, the start time of the local epidemic; *σ*>*t_0_*, the time at which the initial social distancing period began; *p_0_*, the quasistationary fraction of the total population practicing social distancing; *λ_0_*, an eigenvalue characterizing the rate of movement between the mixing and protected subpopulations and establishing a timescale for population-level adoption of social distancing practices; and *β*, which characterized the rate of disease transmission in the absence of social distancing. The measurement model parameter *f_D_* represented the time-averaged fraction of new cases detected. Inference of adjustable parameter values was based on a negative binomial likelihood function (Appendix 1 Equation 27). The dispersal parameter *r* of the likelihood was adjustable; its value was jointly inferred with those of *t_0_*, *σ*, *p_0_*, *λ_0_*, *β*, and *f_D_*.

**Table 1:** Inferred values of parameters in models for forecasting regional epidemics of coronavirus disease, United States

Parameter*	Estimate†	Definition
*t*_0_	33 d	Start of transmission
*σ*	33 d	Start of social distancing
*p*_0_	0.87	Social distancing setpoint
*λ*_0_	0.10/d	Social distancing rate
*β*	2.0/d	Disease transmission rate
*f_D_*	0.12	Fraction of active cases reported
*r*	12	Dispersal parameter of NB(*r*,*p*)‡

*t_0_, *σ*, *p*_0_, *λ*_0_, and *β* are adjustable parameters of the compartmental model; *f_D_* is a parameter of the auxiliary measurement model; and *r* is a parameter for the associated statistical model for noise in case detection and reporting. †All estimates are region-specific and inference-time-dependent. Inferences were conducted daily. These findings reflect the maximum a posteriori estimates inferred for the New York City metropolitan statistical area using all confirmed coronavirus disease case count data available in the GitHub repository maintained by The New York Times newspaper (*11*) for January 21–June 21, 2020. Time *t* = 0 corresponds to midnight on January 21, 2020.  ‡The probability parameter of NB(*r*,*p*) is constrained (i.e., its reporting-time-dependent value is determined by Appendix 1 Equation 26, https://wwwnc.cdc.gov/EID/article/27/3/20-3364-App1.pdf).

**Table 2:** Estimates for the fixed parameters of compartmental model for forecasting regional epidemics of coronavirus disease, United States

Parameter	Estimate	Source
*S_0_*	19,216,182*	US Census Bureau (*13*)
*I_0_*	1	Assumption
*n*	0†	Assumption
*m_b_*	0.1	Assumption
*ρ_E_*	1.1	Arons et al. (*14*)
*ρ_A_*	0.9	Nguyen et al. (*15*)
*k_L_*	0.94/d	Lauer et al. (*12*)
*k_Q_*	0.0038/d	Assumption
*j_Q_*	0.4/d	Assumption
*f_A_*	0.44	(*16*,*17*)
*f_H_*	0.054	Perez-Saez et al. (*18*)
*f_R_*	0.79	Richardson et al. (*19*)
*c_A_*	0.26/d	Sakurai et al. (*17*)
*c_I_*	0.12/d	Wölfel et al. (*20*)
*c_H_*	0.17/d	Richardson et al. (*19*)

*All estimates listed in this table are considered to apply to all regions of interest except for *n*, the number of distinct social distancing periods after an initial social distancing period, and *S_0_*, the region-specific initial number of susceptible persons. The value given here for *S_0_* is the US Census Bureau estimated total population of the New York City metropolitan statistical area.  †*n* = 0, unless stated otherwise.

**Table 3:** Description of the fixed parameters of the compartmental model for forecasting regional epidemics of coronavirus disease, United States

Parameter	Definition
*S_0_*	Initial size of susceptible population*
*I_0_*	Initial no. infected individuals†
*n*	No. prior social distancing periods (e.g., 0 or 1)
*m_b_*	Protective effect of social distancing‡
*ρ_E_*	Relative infectiousness of an exposed person without symptoms during the incubation period§
*ρ_A_*	Relative infectiousness of an asymptomatic person in the immune clearance phase of infection§
*k_L_*	Rate constant for progression through each stage of the incubation period¶
*k_Q_*	Rate constant for entry into quarantine for a person without symptoms
*j_Q_*	Rate constant for entry into quarantine for a person with mild symptoms
*f_A_*	Fraction of all cases that are asymptomatic
*f_H_*	Fraction of all cases of severe disease (including patients requiring hospitalization or isolation at home)
*f_R_*	Fraction of persons with severe disease who eventually recover
*c_A_*	Rate constant for recovery of asymptomatic persons in the immune clearance phase of infection
*c_I_*	Rate constant for recovery of symptomatic persons with mild disease or progression to severe disease#
*c_H_*	Rate constant for recovery of symptomatic persons with severe disease or progression to death**

*Initial susceptible population within a given region is assumed to be the total regional population. †Assuming that there is initially a single infected, symptomatic person. ‡This parameter defines the reduction in disease transmission caused by the protective effects of social distancing. §This parameter characterizes infectiousness relative to a symptomatic person with all other factors being equal (i.e., a symptomatic person exhibiting the same social distancing behavior). ¶The incubation period is divided into 5 stages, each of equal duration on average. #In the model, after a mean waiting time of 1/*c_I_*, symptomatic persons with mild disease recover or progress to severe disease. **In the model, after a mean waiting time of 1/*c_H_*, symptomatic persons with severe disease recover or die.

The compartmental model had 3 adjustable parameters for each additional social distancing period after the initial. For 1 additional period of social distancing (*n* = 1, the additional adjustable parameters were *τ*_1_>*σ*, the onset time of second-phase social distancing; *p*_1_, the second-phase quasistationary setpoint; and *λ*_1_, which determined the timescale for transition from first- to second-phase social distancing behavior. For a second social distancing period, we replaced *p*_0_ with *p*_1_ and *λ*_0_ with *λ*_1_ at time t = *τ*_1_. If adherence to effective social distancing practices began to relax at time t = *τ*_1_, then *p*_1_<*p*_0_.

### Statistical Model for Noisy Case Reporting

We used a deterministic compartmental model to predict the expected number of new confirmed COVID-19 cases reported daily. In other words, we assumed that the number of new cases reported over a 1-day period was a random variable and that the expected value would follow a deterministic trajectory. We further assumed that day-to-day fluctuations in the random variable were independent and characterized by a negative binomial distribution, denoted as NB(*r*,*p*). We used NB(*r*,*p*) to model noise in reporting and case detection. The support of this distribution is the nonnegative integers, which is natural for populations. Furthermore, the shape of NB(*r*,*p*) is flexible enough to recapitulate an array of unimodal empirical distributions. With these assumptions, we obtained a likelihood function (Appendix 1 Equation 27) in the form of a product of probability mass functions of NB(*r*,*p*). Formulation of a likelihood is a prerequisite for standard Bayesian inference; however, some related methods, such as approximate Bayesian computation, do not rely on a likelihood function.

### Online Learning of Model Parameter Values through Bayesian Inference

We used Bayesian inference to identify adjustable model parameter values for each MSA of interest. In each inference, we assumed a uniform prior and used an adaptive Markov chain Monte Carlo algorithm (*21*) to generate samples of the posterior distribution for the adjustable parameters (Appendix 1).

The maximum a posteriori (MAP) estimate of a parameter is the value corresponding to the mode of its marginal posterior, where probability mass is highest. Because we assumed a uniform prior, our MAP estimates were maximum-likelihood estimates.

### Forecasting with Quantification of Prediction Uncertainty: Bayesian Predictive Inference

In addition to inferring parameter values, we quantified uncertainty in predicted trajectories of daily case reports. We obtained a predictive inference of the expected number of new cases detected on a given day by parameterizing a model using a randomly-chosen parameter posterior sample generated in Markov chain Monte Carlo sampling. We then predicted the number of cases detected by adding a noise term, drawn from NB(*r*,*p*), where *r* is set at the randomly sampled value and *p* is set using an equation (Appendix 1 Equation 26).

We used LSODA (*22*; SciPy, https://scipy.org) to numerically integrate the described ODEs and obtain a prediction of the compartmental model for any given (1-day) surveillance period and specified settings for parameter values (Appendix 1 Equations 1–17, 23). The initial condition was defined by the inferred value of *t*_0_ (Table 1) and the fixed settings for *S*_0_ and *I*_0_ (Tables 2, 3). We predicted the actual number of new cases detected by entering the predicted expected number of new cases into an equation (Appendix 1 Equation 29).

The 95% credible interval (CrI) for the predicted number of new case reports on a given day is the central part of the marginal predictive posterior capturing 95% of the probability mass. This region is bounded above by the 97.5th percentile and below by the 2.5th percentile.

## Results

The objective of our study was to detect notable new trends in daily COVID-19 incidence as early as possible. We achieved this goal by systematically and regularly updating mathematical models capturing historical trends in regional COVID-19 epidemics using Bayesian inference and making forecasts with Bayesian uncertainty quantification.

**Figure 2:**
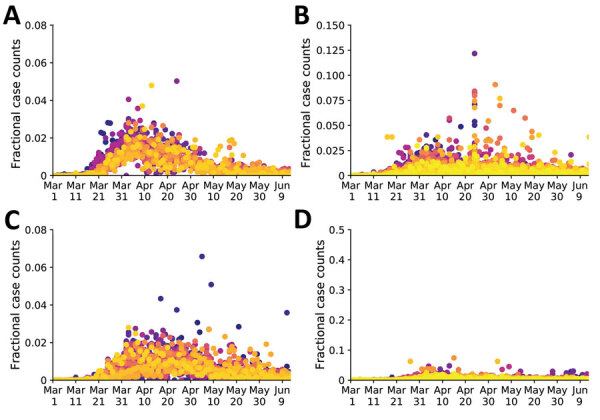
Temporal correlations of fractional case counts of coronavirus disease in and around the New York City, New York, metropolitan statistical area, United States, March 1–June 13, 2020. The fractional case count for a county on a given date is defined as the reported number of cases on that date divided by the total reported number of cases in the county over the entire time period of interest. Panels show the fractional cast counts for: A) the 23 counties comprising the New York City metropolitan statistical area (Fano factor 0.0026); B) the 62 counties comprising New York state (Fano factor 0.021); C) the 21 counties comprising New Jersey (Fano factor 1.2); and D) the 67 counties comprising Pennsylvania (Fano factor 0.028). Within each plot, different colors indicate the data points from each distinct county. Purple–yellow gradient indicates alphabetical order of the counties. A smaller Fano factor indicates less county-to-county variability.

Our analysis focused on the populations of US cities and their MSAs instead of regional populations within other political boundaries, such as those of US states. The boundaries of MSAs are based on social and economic interactions (*10*), which suggests that the population of an MSA is likely to be more uniformly affected by the COVID-19 pandemic than, for example, the population of a state. Accordingly, daily reports of new COVID-19 cases for the New York City MSA (Figure 2, panel A) are more temporally correlated than for the 3 states that make up the New York City MSA: New York (Figure 2, panel B), New Jersey (Figure 2, panel C), and Pennsylvania (Figure 2, panel D). Daily case counts for New Jersey resembled those for New York City because the 2 populations overlap considerably: ≈74% of New Jersey’s population is part of the New York City MSA and ≈32% of the population of the New York City MSA is part of New Jersey (*13*).

**Figure 3:**
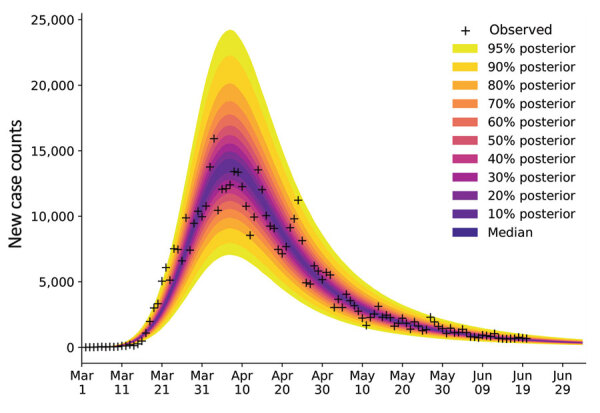
Illustration of Bayesian predictive inference for daily new case counts of coronavirus disease in the New York City, New York, metropolitan statistical area, United States, March 1–June 21, 2020. Daily reports of new cases forecasted with rigorous uncertainty quantification through online Bayesian learning of model parameters. Each day considers all daily case-reporting data available up to that point. We conducted Markov chain Monte Carlo sampling of the posterior distribution for a set of adjustable parameters. Subsampling of the posterior samples enabled the relevant model to generate trajectories of the epidemic curve that account for parametric and observation uncertainty. Crosses indicate observed daily case reports. The shaded region indicates the 95% credible interval for predictions of daily case reports. The color-coded bands within the shaded region indicate alternate credible intervals. The model was parametrized with uncertainty quantification data from January 21–June 21, 2020. The uncertainty bands/inferred model was used to make predictions for 14 days after the last observed data: the last prediction date was July 5, 2020.

For each of the 15 most populous US MSAs, we defined parameters for a compartmental model using MSA-specific surveillance data, namely aggregated county-level reports indicating the number of new confirmed COVID-19 cases within a given MSA each day. We made daily predictions by using Bayesian parameterization and forecasting with uncertainty quantification (UQ) for each of the 15 MSAs (Figure 3). Predictions took the form of a predictive posterior distribution and varied because of the uncertainties in adjustable model parameter estimates, which were characterized quantitatively through Bayesian inference. For these inferences we used the complete time series of available daily new case counts for the region of interest.

**Figure 4:**
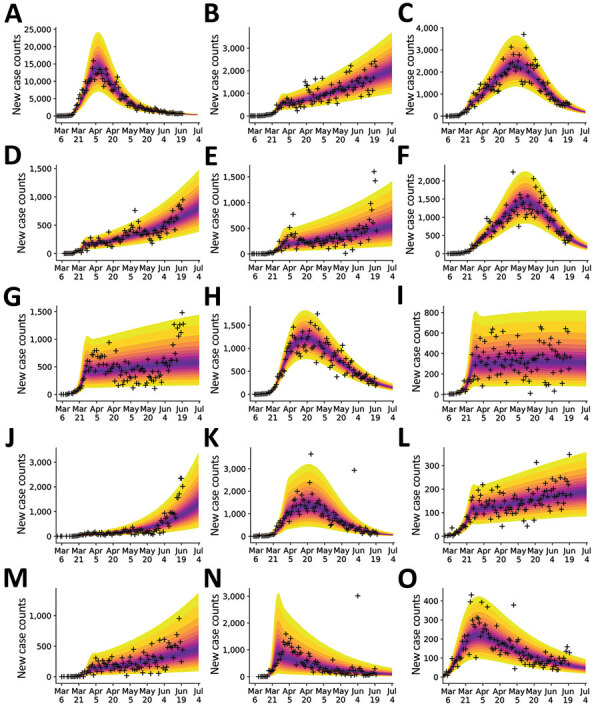
Bayesian predictive inferences for daily new case counts of coronavirus disease in the 15 most populous metropolitan statistical areas, United States, March 1–June 21, 2020. Predictions conditioned on the compartmental model with structure defined by *n* = 0, which accounts for a single initial period of social distancing. Inferences shown for the metropolitan statistical areas for the following cities: A) New York City, New York; B) Los Angeles, California; C) Chicago, Illinois; D) Dallas, Texas; E) Houston, Texas; F) Washington, DC; G) Miami, Florida; H) Philadelphia, Pennsylvania; I) Atlanta, Georgia; J) Phoenix, Arizona; K) Boston, Massachusetts; L) San Francisco, California; M) Riverside, California; N) Detroit, Michigan; and O) Seattle, Washington. Crosses indicate observed daily case reports. The shaded region indicates the 95% credible interval for predictions of daily case reports. The color-coded bands within the shaded region indicate alternate credible intervals. The model was parametrized with uncertainty quantification by using data from January 21–June 21, 2020. The uncertainty bands/inferred model was used to make predictions for 14 days after the last observed data: the last prediction date was July 5, 2020.

We conducted predictive inferences for all 15 MSAs of interest (Figure 4). We conditioned our predictions on the compartmental model with *n* = 0. These results demonstrate that, for the timeframe of interest, the compartmental model with *n* = 0 can reproduce many of the empirical epidemic curves for the MSAs of interest, which vary in shape.

**Figure 5:**
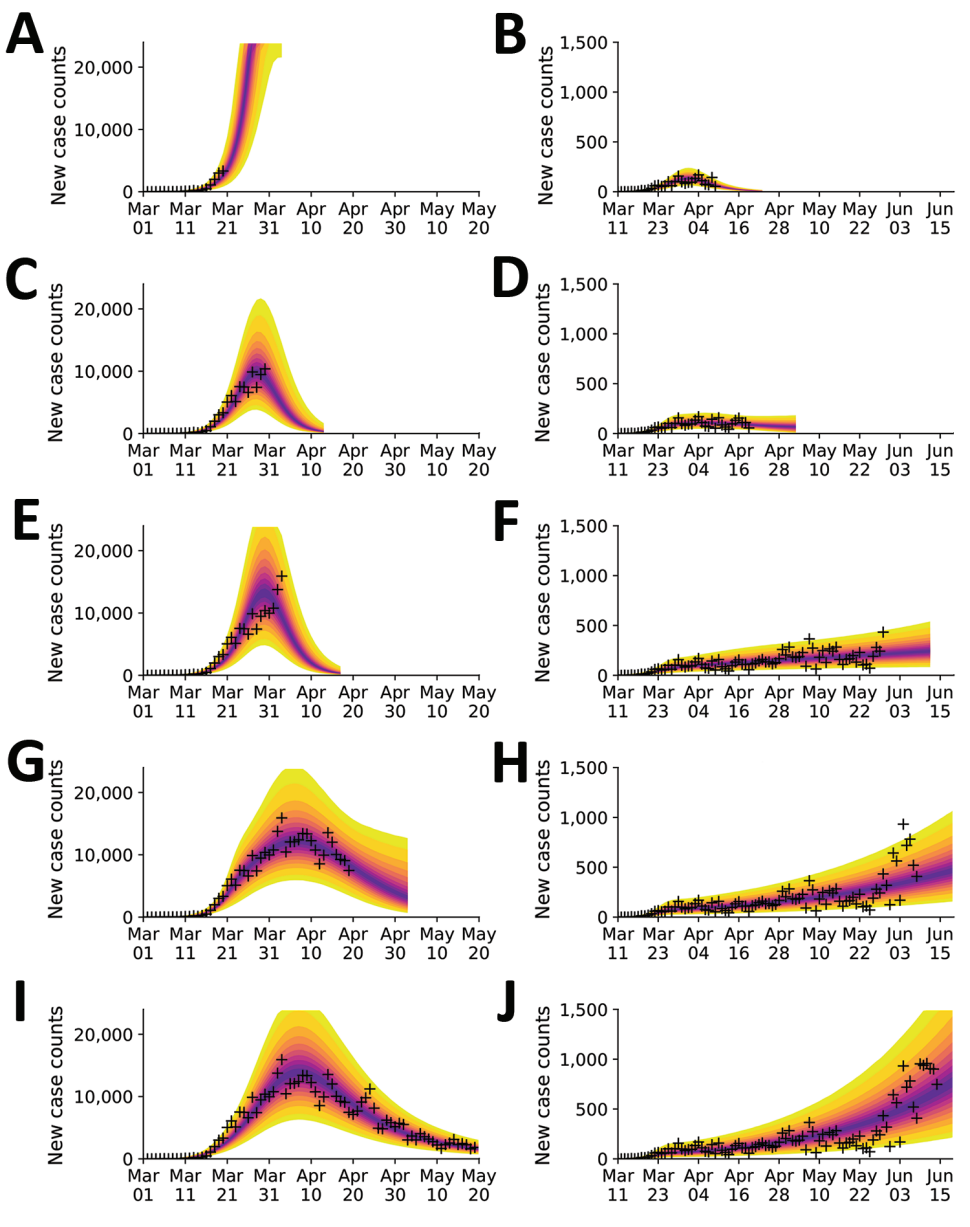
Illustration of the need for online learning for modeling daily new case counts of coronavirus disease in the New York City, New York, and Phoenix, Arizona, metropolitan statistical areas, United States, 2020. Predictions made over a series of progressively later dates as indicated for the New York City area (A, C, E, G, I) and the Phoenix area (B, D, F, H, J). Predictive inferences are data driven and conditioned on a compartmental model. Crosses indicate observed daily case reports. The shaded region indicates the 95% credible interval for predictions of daily case reports. The color-coded bands within the shaded region indicate alternate credible intervals. Predictions are accurate but only over a finite period of time into the future. New data must be considered as these data become available to maintain prediction accuracy. The model was parametrized with uncertainty quantification using all data up to a terminal date, which differs in each panel. The uncertainty bands/inferred model was used to make predictions for 14 days after the last observed data point. For the New York City area, visualization began on March 1, 2020; the terminal dates were A) March 20, C) March 30, E) April 3, G) April 19, and I) May 19, 2020. For the Phoenix area, visualization began on March 11, 2020; the terminal dates were B) April 9, D) April 19, F) May 29, H) June 8, and J) June 18, 2020.

We also calculated predictive inferences for the New York City and Phoenix MSAs over time (Figure 5; Appendix 2 Videos 1, 2). These results illustrate that accurate short-term predictions are possible; however, continual updating of parameter estimates is required to maintain accuracy.

**Figure 6:**
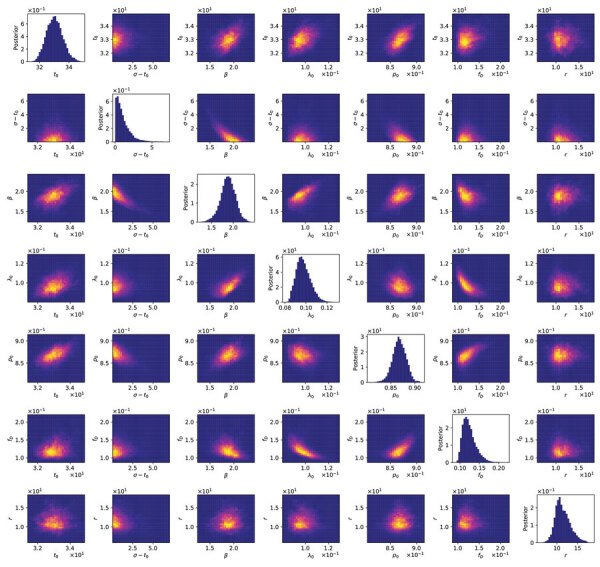
Matrix of 1- and 2-dimensional projections of the 7-dimensional posterior samples obtained for the adjustable parameters associated with the compartmental model (*n* = 0) for daily new case counts of coronavirus disease in the New York City, New York, metropolitan statistical area, United States, January 21–June 21, 2020. Plots of marginal posteriors (1-dimensional projections) are shown on the diagonal from top left to bottom right. Other plots are 2-dimensional projections indicating the correlations between parameter estimates. Brightness indicates higher probability density. A compact bright area indicates absence of or relatively low correlation. An extended, asymmetric bright area indicates relatively high correlation.

We found that the adjustable parameters of the compartmental model had identifiable values, meaning that their marginal posteriors were unimodal (Figure 6). In the context of a deterministic model, the significance of identifiability is that, despite uncertainties in parameter estimates, we can expect predictive inferences of daily new case reports to cluster around a central trajectory. The results are representative (Figure 6); we routinely recovered unimodal marginal posteriors. However, we do not have a mathematical proof of identifiability for our model.

Usually, when we forecasted with UQ, the empirical new case count for the day immediately following our inference (+1), and often for each of several additional days, fell within the 95% CrI of the predictive posterior. When the reported number of new cases falls outside the 95% CrI and above the 97.5 percentile, we interpret this upward-trending rare event to have a probability of <0.025, assuming the model is both explanatory (i.e., consistent with historical data) and predictive of the near future. If the model is predictive of the near future, the probability of 2 consecutive rare events is far smaller, <0.001. Thus, consecutive upward-trending rare events, called upward-trending anomalies, can indicate that the model is not predictive. An anomaly suggests that the rate of COVID-19 transmission has increased beyond what can be explained by the model.

**Figure 7:**
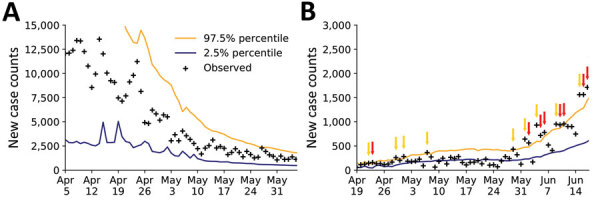
Rare events and anomalies in daily new case counts of coronavirus disease in (A) the New York City, New York metropolitan statistical area during April 5–June 4, 2020 and (B) Phoenix, Arizona, metropolitan statistical area during April 19–June 18, 2020, United States. Crosses indicate observed daily case reports. Orange line indicates 97.5% probability percentile; blue line indicates 2.5% probability percentile. Yellow arrows mark upward-trending rare events. Red arrows mark upward-trending anomalies.

We did not observe upward-trending anomalies for the New York City MSA (Figure 7, panel A). However, for the Phoenix MSA, we observed several anomalies that preceded rapid and sustained growth in the number of new cases reported per day in June (Figure 7, panel B).

**Figure 8:**
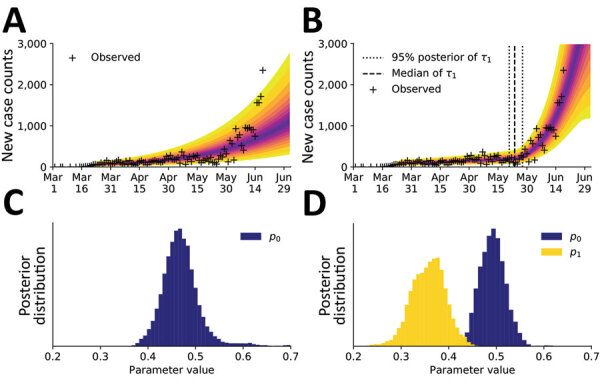
Predictions of the compartmental model for daily new case counts of coronavirus disease in the Phoenix, Arizona, metropolitan statistical area, United States, January 21–June 18, 2020. A) Model using 1 initial period of social distancing (*n* = 0). B) Model using an initial period of social distancing and a subsequent period of reduced adherence to social distancing practices (*n* = 1). C) The marginal posteriors for the social-distancing setpoint parameter *p*_0_ inferred in panel A. D) The marginal posteriors for the social-distancing parameters *p*_0_ and *p*_1_ inferred in panel B.

We assumed these anomalies arose from behavioral changes. To explain them, we enabled the compartmental model to account for a second social distancing period by increasing the setting for *n* from 0 to 1. With this change, the number of adjustable parameters increased from 7 to 10. One of the new parameters was *τ*_1_, the start time of the second social distancing period. The other new parameters, *λ*_1_ and *p*_1_, replaced *λ*_0_ and *p*_0_ at time t = *τ*_1_ The compartmental model with 2 social distancing periods better explained the data from Phoenix than the compartmental model with only 1 social distancing period (Figure 8, panels A and B). This conclusion is supported by the Akaike and Bayesian information criteria values for the 2 scenarios (Appendix 1 Table 1). Although these criteria are crude model selection tools in the context of non-Gaussian posteriors, we decided that they were adequately discriminatory. Each strongly indicates that the model with 2 social distancing periods better represented the data than the model with 1 social distancing period. Furthermore, the MAP estimate for *p*_1_ (≈0.38) was less than that for *p*_0_ (≈0.49) (Figure 8, panels C, D) and the marginal posteriors for these parameters were largely nonoverlapping (Figure 8, panel D). These findings suggest that the increase in COVID-19 cases in Phoenix can be explained by relaxation in social distancing practices, quantified by our estimates for *p*_0_ and *p*_1_. The MAP estimate of the start time of the second period of social distancing corresponds to May 24, 2020 (95% CrI May 20–28, 2020). Overall, 8 of the 9 observed anomalies occurred after this period, the first of which occurred on June 2, 2020 (Figure 8, panel B).

We hypothesized that a single event generating thousands of new infections, such as a mass gathering, might prompt a new upward trend in COVID-19 transmission. However, simulations for New York City and Phoenix did not support this hypothesis (Appendix 1 Figure 2). In each of these simulations, we moved a specified number of persons from the mixing susceptible population *S_M_* into the exposed population *E*_1_ at the indicated time, May 30, 2020. Each perturbation increased disease incidence but had minimal effect on the slope of the trajectory of new case detection.

In addition to Phoenix, 4 other MSAs had contemporaneous trends explainable by relaxation of social distancing (Appendix 1 Table 1, Figure 3). MAP estimates for *τ*_1_ indicate that the second social distancing period began on May 27, 2020 in Houston; April 19, 2020 in Miami; May 24, 2020 in Phoenix; June 12, 2020 in San Francisco; and June 7, 2020 in Seattle (Appendix 1 Figure 3). We detected upward-trending anomalies for these 5 MSAs (Appendix 1 Figure 4, panels A–D), but not for 3 of 4 other MSAs that had epidemic curves consistent with sustained social distancing (Appendix 1 Figure 4, panels E–H; Appendix 2 Videos 3–10). We assessed the overall prediction accuracy of the region-specific compartmental models (Appendix 1 Figure 5).

## Discussion

We found that online learning of model parameter values from real-time surveillance data is feasible for mathematical models of COVID-19 transmission. Furthermore, we found that predictive inference of the daily number of new cases reported is feasible for regional COVID-19 epidemics occurring in multiple US MSAs. We are continuing to perform daily forecasts and to disseminate the results (*23*,*24*). Inferences are computationally expensive and the cost increases as new data become available; thus, daily inferences using these methods might be impractical in some circumstances.

These predictive inferences can be used to identify harbingers of future growth in COVID-19 transmission rates. We found that 2 consecutive upward-trending rare events in which the number of new cases reported is above the upper limit of the 95% CrI of the predictive posterior might indicate potential for increased transmission during the following days to weeks. This feature might be especially predictive when anomalies are accompanied by increasing prediction uncertainty, as seen in Phoenix (Figure 7, panel B).

We found that the June increase in transmission rate of COVID-19 in the Phoenix metropolitan area can be explained by a reduction in the percentage of the population adhering to effective social distancing practices from ≈49% to ≈38% (Figure 8, panel D). However, our study sheds no light on which social distancing practices are effective at slowing COVID-19 transmission. We inferred that relaxation of social distancing measures began around May 24, 2020 (Figure 8, panel B). Contemporaneous upward trends in the rate of COVID-19 transmission in the Houston, Miami, San Francisco, and Seattle MSAs can also be explained by relaxation of social distancing (Appendix 1 Table 1, Figure 3). These findings are qualitatively consistent with earlier studies indicating that social distancing is effective at slowing the transmission of COVID-19 (*7*,*8*). These results also suggest that the future course of the pandemic is controllable, especially with accurate recognition of when stronger nonpharmaceutical interventions are needed to slow COVID-19 transmission.

One limitation of our study is that trend detection is data-driven, which means that a new trend cannot be detected until enough evidence has accumulated. Our analysis used reports of new cases, which reflect transmission dynamics of the past days to weeks rather than the current moment. Other types of surveillance data, such as assays of viral RNA in wastewater samples, also might improve situational awareness. Another limitation is that our inferences are based on a mathematical model associated with considerable structure and fixed parameter uncertainties and simplifications. Among the simplifications is the replacement of certain time-varying parameters, such as those characterizing testing capacities, with constants, which are assumed to provide an adequate time-averaged characterization. In this study, we used a deterministic compartmental model. If disease prevalence decreases, a stochastic version of the model might be more appropriate for forecasting efforts. Although the model can reproduce historical data and make accurate short-term forecasts, its structure and fixed parameters are subject to revision as we learn more about COVID-19. Furthermore, the model will need to be revised to account for vaccination. Results from serologic studies and estimates of excess deaths should enable model improvements.

Appendix 1 Further information on daily forecasting of regional epidemics of coronavirus disease in the United States with Bayesian uncertainty quantification.

Appendix 2 Videos for daily forecasting of regional epidemics of coronavirus disease in the United States with Bayesian uncertainty quantification
