# Supplementary material for: Daily Forecasting of Regional Epidemics of Coronavirus Disease with Bayesian Uncertainty Quantification, United States
Source: Emerg Infect Dis. 2021 Mar;27(3):767–78. doi: 10.3201/eid2703.203364 (PMC7920670; doi:10.3201/eid2703.203364)
Supplement: Appendix 1 — Further information on daily forecasting of regional epidemics of coronavirus disease in the United States with Bayesian uncertainty quantification. [file 20-3364-Techapp-s1.pdf]

Article DOI: https://doi.org/10.3201/eid2703.203364

Daily Forecasting of Regional Epidemics of Coronavirus Disease with Bayesian Uncertainty Quantification, United States

Appendix

Full Description of the Mechanistic Compartmental Model

The compartmental model (Appendix Figure 1), consists of the following 25 ordinary differential equations (ODEs):

$$\frac{dS_M}{dt} = -\beta\left(\frac{S_M}{S_0}\right)(\phi_M(t,\rho) + m_b\phi_P(t,\rho)) - U_\sigma(t)\Lambda_\tau(t)[P_\tau(t)S_M - (1 - P_\tau(t))S_P] \quad [1]$$

$$\frac{dS_P}{dt} = -m_b\beta\left(\frac{S_P}{S_0}\right)(\phi_M(t,\rho) + m_b\phi_P(t,\rho)) + U_\sigma(t)\Lambda_\tau(t)[P_\tau(t)S_M - (1 - P_\tau(t))S_P] \quad [2]$$

$$\frac{dE_{1,M}}{dt} = \beta\left(\frac{S_M}{S_0}\right)(\phi_M(t,\rho) + m_b\phi_P(t,\rho)) - k_L E_{1,M} - U_\sigma(t)\Lambda_\tau(t)\left[P_\tau(t)E_{1,M} - (1 - P_\tau(t))E_{1,P}\right] \quad [3]$$

$$\frac{dE_{1,P}}{dt} = m_b\beta\left(\frac{S_P}{S_0}\right)(\phi_M(t,\rho) + m_b\phi_P(t,\rho)) - k_L E_{1,P} + U_\sigma(t)\Lambda_\tau(t)\left[P_\tau(t)E_{1,M} - (1 - P_\tau(t))E_{1,P}\right] \quad [4]$$

$$\frac{dE_{i,M}}{dt} = k_L E_{i-1,M} - k_L E_{i,M} - k_Q E_{i,M} - U_\sigma(t)\Lambda_\tau(t)\left[P_\tau(t)E_{i,M} - (1 - P_\tau(t))E_{i,P}\right], \quad [5]$$

for $i = 2, 3, 4, 5$

$$\frac{dE_{i,P}}{dt} = k_L E_{i-1,P} - k_L E_{i,P} - k_Q E_{i,P} + U_\sigma(t)\Lambda_\tau(t)\left[P_\tau(t)E_{i,M} - (1 - P_\tau(t))E_{i,P}\right], \quad [6]$$

for $i = 2, 3, 4, 5$

$$\frac{dE_{2,Q}}{dt} = k_Q(E_{2,M} + E_{2,P}) - k_L E_{2,Q} \quad [7]$$

$$\frac{dE_{i,Q}}{dt} = k_Q\left(E_{i,M} + E_{i,P}\right) + k_L E_{i-1,Q} - k_L E_{i,Q}, for\ i = 3, 4, 5 \quad [8]$$

$$\frac{dA_M}{dt} = f_A k_L E_{5,M} - k_Q A_M - U_\sigma(t)\Lambda_\tau(t)[P_\tau(t)A_M - (1 - P_\tau(t))A_P] - c_A A_M \quad [9]$$

$$\frac{dA_P}{dt} = f_A k_L E_{5,P} - k_Q A_P + U_\sigma(t)\Lambda_\tau(t)[P_\tau(t)A_M - (1 - P_\tau(t))A_P] - c_A A_P \quad [10]$$

$$\frac{dA_Q}{dt} = f_A k_L E_{5,Q} + k_Q(A_M + A_P) - c_A A_Q \quad [11]$$

$$\frac{dI_M}{dt} = (1 - f_A)k_L E_{5,M} - \left(k_Q + j_Q\right)I_M - U_\sigma(t)\Lambda_\tau(t)\left[P_\tau(t)I_M - \left(1 - P_\tau(t)\right)I_P\right] - c_I I_M \quad [12]$$

$$\frac{dI_P}{dt} = (1 - f_A)k_L E_{5,P} - \left(k_Q + j_Q\right)I_P + U_\sigma(t)\Lambda_\tau(t)[P_\tau(t)I_M - \left(1 - P_\tau(t)\right)I_P] - c_I I_P \quad [13]$$

$$\frac{dI_Q}{dt} = (1 - f_A)k_L E_{5,Q} + \left(k_Q + j_Q\right)(I_M + I_P) - c_I I_Q \quad [14]$$

$$\frac{dH}{dt} = f_H c_I\left(I_M + I_P + I_Q\right) - c_H H \quad [15]$$

$$\frac{dD}{dt} = (1 - f_R)c_H H \quad [16]$$

$$\frac{dR}{dt} = c_A\left(A_M + A_P + A_Q\right) + (1 - f_H)c_I\left(I_M + I_P + I_Q\right) + f_R c_H H \quad [17]$$

where β, S_0, m_b, k_L, k_Q, j_Q, f_A, f_H, f_R, c_A, c_I, and c_H are positive-valued time-invariant parameters (Tables 1, 3). Parameter names are unique but only within the namespace of a given model. Each ODE in equations 1–17 defines the time-rate of change of a subpopulation (i.e., the time-rate of change of a state variable). There are 25 state variables, 1 for each ODE. Equation 5 defines 4 ODEs, 6 defines 4, and 8 defines 3 ODEs of the model. The model does not include new cases caused by travel.

The initial condition is $S_M(t_0) = S_0$, $I_M(t_0) = I_0 = 1$, with all other populations (S_P, $E_{1,M}, \dots, E_{5,M}$, $E_{1,P}, \dots, E_{5,P}$, $E_{2,Q}, \dots, E_{5,Q}$, A_M, A_P, A_Q, I_P, I_Q, H, D, and R) equal to 0. The

parameter S_0 denotes the total region-specific population size. Thus, we assume that the entire population is susceptible at the start of the epidemic at time $t = t_0$>0, where time $t = 0$ is 00:00 hours on January 21, 2020. The parameter I_0, which we always consider to be 1, denotes the number of infectious symptomatic persons at the start of the regional epidemic.

Subscripts attached to state variables are used to denote subpopulations. The subscript M represents mixing populations and P represents protected populations. For example, the variables S_M and S_P denote the population sizes of mixing and protected persons who are susceptible to infection. Persons in a protected population practice social distancing; persons in a mixing population do not. The approach that we have taken to model social distancing is similar to that of Anderson et al. (S. Anderson, unpub. data, https://www.medrxiv.org/content/10.1101/2020.04.17.20070086v1).

The incubation period is divided into 5 stages. The numerical subscripts 1, 2, 3, 4, and 5 attached to E variables indicate progression through these 5 stages. Exposed persons in the incubation period, except for those in the first stage, are considered to be infectious but without symptoms. They are either presymptomatic (i.e., will later have symptoms) or asymptomatic (i.e., will never have symptoms).

The subscript Q is attached to variables representing populations of quarantined persons. The state variable I_Q is a special case; it accounts for symptomatic persons who are quarantined as well as persons who are self-isolating because of symptom awareness.

The parameter k_Q characterizes the rate at which infected persons move into quarantine because of testing and contact tracing. The parameter j_Q characterizes the rate at which symptomatic persons self-isolate because of symptom awareness. We recognize that susceptible persons may enter quarantine (through contact tracing) but we assume that the size of the quarantined population is negligible compared to that of the total susceptible population and that susceptible persons entering quarantine leave quarantine as susceptible persons.

The parameters β and $m_b < 1$ characterize transmission of disease: β characterizes the rate of transmission attributable to contacts between 2 mixing persons, $m_b\beta$ characterizes the rate of transmission attributable to contacts between 1 mixing and 1 protected person, and $m_b^2\beta$ characterizes the rate of transmission attributable to contacts between 2 protected persons.

Infectious persons considered to contribute to coronavirus disease (COVID-19) transmission include those in the following pools: $E_{2,M}, \ldots, E_{5,M}$ and $E_{2,P}, \ldots, E_{5,P}$, A_M and A_P, and I_M and I_P. We do not consider persons in the first stage of the incubation period (i.e., persons in E_1 pools) to be infectious because we assume these persons are not shedding enough virus to be infectious or detectable in surveillance testing. In experiments with an animal model (the golden hamster, *Mesocricetus auratus*), infectious virus could be recovered from animals 2 days post-inoculation (*2*). Moreover, it was found that severe acute respiratory syndrome coronavirus 2 (SARS-CoV-2) could be detected in contacts of infected animals just 1 day post-contact (*2*). Kucirka et al. (*3*) estimated that the false negative rate for nasal samples from exposed persons tested for SARS-CoV-2 infection an estimated 1 day after exposure is 100% but <100% thereafter. Thus, it seems reasonable to assume that exposed persons beyond the first incubation stage, which has a duration of ≈1 day (on the basis of our estimate for k_L, which is discussed below), are infectious and may be detected as such.

The variables $E_{1,M}, \ldots, E_{5,M}$ and $E_{1,P}, \ldots, E_{5,P}$ denote the population sizes of mixing and protected exposed persons in the 5 stages of the incubation period. The variables $E_{2,Q}, \ldots, E_{5,Q}$ denote the population sizes of quarantined exposed persons in the 5 stages. There is no $E_{1,Q}$ population, as we assume that persons in the first stage of the incubation period are unlikely to test positive for SARS-CoV-2 or to be reached in contact tracing efforts before leaving the E_1 state. The parameter k_L characterizes disease progression, from 1 stage of the incubation period to the next and ultimately to an immune clearance phase. Persons leaving the E_5 pools enter the immune clearance phase, meaning that they become eligible for recovery. Any person leaving an E_5 pool with symptom onset enters an *I* pool, whereas an person leaving an E_5 pool without symptom onset enters an *A* pool. Persons in I pools are considered to have mild disease with the possibility to progress to severe disease.

The dynamics of social distancing are characterized by 3 step functions (i.e., piecewise constant functions having only finitely many pieces): U_σ, Λ_τ, and P_τ. The subscripts attached to these functions denote times: σ is a particular time, whereas τ is a set of times, as discussed later. The value of U_σ switches from 0 to 1 at time $t = \sigma > t_0$, the start of an initial social distancing period. As discussed later, the function Λ_τ defines a timescale for change in social distancing practices for one or more distinct periods of social distancing, and the function P_τ establishes a

setpoint for the fraction of the total population of susceptible and infectious persons adhering to social distancing practices for $\geq$1 distinct periods of social distancing. This population of persons adhering to social distancing practices excludes those persons who are quarantined, self-isolated, and hospitalized.

The parameter f_A denotes the fraction of infected persons who remain asymptomatic. The variables A_M and A_P denote the sizes of the populations of mixing and protected persons who have been infected, progressed through the incubation period, are currently in the immune clearance phase, and will never develop symptoms. The parameter c_A characterizes the rate at which asymptomatic persons recover. Note that the duration of the immune clearance phase for asymptomatic persons, $\hat{t}_A$, is distributed according to $e^{-c_A \hat{t}_A}$ and the mean value of $\hat{t}_A$ is $1/c_A$.

The variable R tracks recoveries of asymptomatic persons, symptomatic persons with mild disease, and hospitalized symptomatic persons with severe disease. All persons who recover are assumed to have immunity, an assumption that is supported by the finding that SARS-CoV-2 infection elicits functional T-cell memory (*4*). Moreover, neutralizing antibodies evidently protect against SARS-CoV-2 infection (*5*). Reinfection has been detected (*6*) but the implications of this apparently rare phenomenon have yet to be determined.

The variables I_M and I_P denote the sizes of the populations of mixing and protected symptomatic persons with mild disease. The parameter c_I characterizes the rate at which symptomatic persons with mild disease recover or progress to severe disease. The parameter f_H is the fraction of symptomatic persons who progress to severe disease requiring hospitalization. As a simplification, we assume that all persons with severe disease are hospitalized or isolated at home in an equivalent state. The duration of the immune clearance phase for symptomatic persons who never progress to severe disease, $\hat{t}_I$, is distributed according to $e^{-c_I \hat{t}_I}$. The mean value of $\hat{t}_I$ is $1/c_I$. As is implicit in our definition of c_I, the time required for progression from mild to severe disease is considered the same as the recovery time of symptomatic persons who experience only mild disease.

The variable H represents the population of hospitalized or severely ill persons. In the model, these persons are considered to be quarantined. Thus, the model does not consider nosocomial transmission. The parameter f_R denotes the fraction of hospitalized severely ill persons who recover. The parameter c_H characterizes the hospital discharge rate, i.e., the rate at

which hospitalized persons with severe disease either recover or die. The variable D tracks deaths. Many deaths occur outside a hospital setting (I. Papst, unpub. data, https://www.medrxiv.org/content/10.1101/2020.09.01.20186395v2). As a simplification, the model does not distinguish between deaths at home and deaths in a hospital. Of note, the mean duration of the immune clearance phase for hospitalized or severely ill persons who recover, $\hat{t}_H$, is distributed according to $\left(e^{-c_I \hat{t}_H} - e^{-c_H \hat{t}_H}\right) c_I c_H / (c_H - c_I)$, assuming $c_H > c_I$. The mean value of $\hat{t}_H$ is $1/c_I + 1/c_H$. As is implicit in our definition of c_H, the time required for progression from severe disease to death is considered to be the same as the recovery time of hospitalized or severely ill persons.

The time-dependent terms $\phi_M(t, \rho)$ and $\phi_P(t, \rho)$ appearing in equations 1–4 represent the effective population sizes of infectious persons in the mixing and protected subpopulations, respectively. These quantities are defined as follows:

$$\phi_M(t, \rho) \equiv I_M + \rho_E\left(E_{2,M} + E_{3,M} + E_{4,M} + E_{5,M}\right) + \rho_A A_M \qquad [18]$$

$$\phi_P(t, \rho) \equiv I_P + \rho_E\left(E_{2,P} + E_{3,P} + E_{4,P} + E_{5,P}\right) + \rho_A A_P \qquad [19]$$

where $\rho = (\rho_E, \rho_A)$, ρ_E is a constant characterizing the relative infectiousness of presymptomatic persons compared to symptomatic persons (with the same behaviors) and ρ_A is a constant characterizing the relative infectiousness of asymptomatic persons compared to symptomatic persons (with the same behaviors). Recall that infectiousness due to social distancing behaviors is captured in equations 1 and 2. Further recall that we assume that persons in the first stage of the incubation period (i.e., persons in either the $E_{1,M}$ or $E_{1,P}$ population) are not infectious. We also assume that the persons in these populations cannot be quarantined until after transitioning to the $E_{2,M}$ or $E_{2,P}$ population because they are assumed to test negative and because contact tracing is assumed to be too slow to catch persons in the transient first stage of incubation. Recall that persons in the A_M, A_P, and A_Q populations are defined as persons who became infected, passed through all 5 stages of the incubation period, are currently in the immune clearance phase, and will never have symptoms. Thus, persons in the exposed E populations include both presymptomatic persons (i.e., persons who will enter the I populations) and asymptomatic persons (i.e., persons who will enter the A populations).

The time-dependent terms U_σ(t), $P_\tau(t)$, and $\Lambda_\tau(t)$ appearing in equations 1–6, equations 9 and 10, and equations 12 and 13 are step functions defined as follows:

$$U_\sigma(t) = \begin{cases} 0 & t < \sigma \\ 1 & t \geq \sigma \end{cases} \quad [20]$$

$$P_\tau(t) = \begin{cases} p_0 & \sigma \leq t < \tau_1 \\ p_1 & \tau_1 \leq t < \tau_2 \\ \vdots & \vdots \\ p_n & \tau_n \leq t < \infty \end{cases} \quad [21]$$

$$\Lambda_\tau(t) = \begin{cases} \lambda_0 & \sigma \leq t < \tau_1 \\ \lambda_1 & \tau_1 \leq t < \tau_2 \\ \vdots & \vdots \\ \lambda_n & \tau_n \leq t < \infty \end{cases} \quad [22]$$

where $\sigma > t_0$ is the time at which widespread social distancing initially begins, the integer $n \geq 0$ is the number of societal (major or widespread) shifts in social-distancing practices after the initial onset of social distancing, each $p_i < 1$ is a parameter characterizing the quasistationary fraction of susceptible persons practicing social distancing during the $(i + 1)$th period of social distancing, each λ_i is a constant defining a timescale for change in social-distancing practices during the $(i + 1)$th period of social distancing, $\tau = \{\tau_0, \dots, \tau_{n+1}\}$, $\tau_0 \equiv \sigma$, $\tau_{n+1} \equiv \infty$, and $\tau_{i+1} > \tau_i$ for $i = 0, \dots, n - 1$. The value of $P_\tau(t)$ defines a setpoint for the quasistationary size of the protected population of susceptible persons, $P_\tau(t) \times 100\%$ of the total susceptible population. The value of $\Lambda_\tau(t)$ determines how quickly the setpoint is reached. As indicated in equations 21 and 22, we only consider step-changes in the values of $P_\tau(t)$ and $\Lambda_\tau(t)$, a simplification. Thus, for a period during which social-distancing practices are intensifying (relaxing), we increase (decrease) the value of $P_\tau(t)$ at the start of the period in a step-change and then hold it constant until the next step-change, if any. Note that σ is the start time of the initial social-distancing period. The time at which the initial social-distancing setpoint, determined by p_0, is reached occurs later and is determined by λ_0, which should not to be confused with the setpoint parameters $p_0, p_1, \dots, p_n$ with the distributional parameter p in the negative binomial distribution NB(r, p).

Full Description of the Auxiliary Measurement Model

To determine how consistent each parameterization of the compartmental model is with available COVID-19 surveillance data, we had to define a quantity—a model output—that corresponds to daily reports of the number of new confirmed COVID-19 cases. Case reporting by public health officials is typically daily. We expected that most cases were detected because of symptom driven (rather than random) testing, visits to a clinical setting, or both. Accordingly, as a simplification, we assumed that persons detected in surveillance are symptomatic. To define a model output comparable to the number of new cases reported on a given day, we started by considering the predicted cumulative number of presymptomatic persons who could become symptomatic while evading quarantine (because of contact tracing) until at least the onset of symptoms, which we will denoted as C_S. According to the model, the time rate of change of C_S is given by the following equation:

$$\frac{dC_S}{dt} = (1 - f_A)k_L(E_{5,M} + E_{5,P}) \qquad [23]$$

The right-hand side of this equation gives the rate at which nonquarantined presymptomatic persons exit the incubation period and enter the immune clearance phase, in which they are symptomatic and therefore considered detectable in local surveillance efforts. We assumed that symptomatic persons in quarantine make a negligible contribution to detection of new cases.

Equation 23 and the ODEs of the compartmental model form a coupled system of equations, which can be numerically integrated to obtain trajectories for the state variables and C_S, the expected cumulative number of symptomatic cases. From the trajectory for C_S, we obtain a prediction for $I(t_i, t_{i+1})$, the expected number of new COVID-19 cases reported on a given calendar date $\mathcal{D}_\mathrm{i}$, from the following equation:

$$I(t_i, t_{i+1}) = f_D[C_S(t_{i+1}) - C_S(t_i)] \qquad [24]$$

where f_D is an adjustable region-specific parameter characterizing the time-averaged fraction of symptomatic cases detected among nonquarantined and hospitalized persons. Equation 24 completes the formulation of our measurement model. $I(t_i, t_{i+1})$ is the model output that we compare to δC_i, the number of new cases reported on calendar date $\mathcal{D}_\mathrm{i}$.

Adjustable and Fixed Parameters of the Compartmental and Auxiliary Measurement Models

The parameters of the compartmental model (equations 1–22) and the auxiliary measurement model (equations 23 and 24) are considered to have either adjustable or fixed values. The adjustable parameter values were estimated (daily) through Bayesian inference on the basis of surveillance data (i.e., reports of newly detected cases). The fixed parameter values are held constant during inference and are based on nonsurveillance data, assumptions, or both, which are discussed in the section below. In this section, we simply delineate the parameters with adjustable and fixed values. The compartmental model formulated for a given regional epidemic has a total of $16 + 3(n + 1)$ parameters. The value of n is structural; it sets the number social-distancing periods considered.

The value of n corresponds to the number of periods of distinct social-distancing behaviors that follow an initial period of social distancing, which we take to begin at time $t = \sigma > t_0$. Here, we take $n = 0$ or 1 for all regional epidemics of interest. Initially, we set $n = 0$. In cases where we set $n = 1$, this setting was motivated by second wave-type dynamics suggested by the surveillance data, which we take to indicate a relaxation of social distancing practices at time $t = \tau_1 > \sigma$. The parameters of the initial social distancing period are σ, p_0, and λ_0. The parameters of the second social distancing period, if considered, are τ_1, p_1, and λ_1. Thus, there are $3(n + 1)$ social-distancing parameters, all of which were adjustable.

In addition to the $3(n + 1)$ social distancing parameters, we have 16 other parameters. Of these, 3 define the initial condition: t_0, $S_M(t = t_0) = S_0$, and $I_M(t = t_0) = I_0$, where t_0 is the time at which the epidemic begins, S_0 is the total population of the region of interest, and I_0, the initial number of infected persons, is always assumed to be 1. We take t_0 to be adjustable and S_0 and I_0 to be fixed. The value of S_0 is set on the basis of population estimates by the US Census Bureau for the metropolitan statistical areas of interest (*7*), which are delineated by the US Office of Management and Budget (*8*).

The final adjustable parameter of the compartmental model, β, characterizes the rate of disease transmission attributable to contacts among persons within the mixing population. In the period before the onset of social distancing, from t_0 to σ, when $S_M/S_0 \approx 1$, the instantaneous rate of disease transmission is $\beta\phi_M(t, \rho)$, where $\phi_M(t, \rho)$ is the effective number of infectious

persons at time t, a weighted sum of the numbers of symptomatic, presymptomatic, and asymptomatic persons determined by $\rho = (\rho_E, \rho_A)$. We assumed that exposed persons after the first stage of disease incubation are infectious, as are asymptomatic persons in the immune clearance phase who have passed through all 5 stages of disease incubation and who will never develop symptoms.

The remaining 12 parameters of the compartmental model, which are considered to have fixed, region-independent values, are as follows: m_b, ρ_E, ρ_A, k_L, k_Q, j_Q, f_A, f_H, c_A, c_I, f_R, and c_H. Our estimates for these parameters are discussed in the section immediately below. Of note, settings for f_R and c_H do not affect predictions of new cases because these parameters characterize recovery or morbidity of hospitalized persons. The parameter f_R is the fraction of hospitalized persons who recover, and the parameter c_H characterizes the hospital discharge rate. Although nosocomial disease transmission is a significant concern, we assume that hospitalized persons are effectively quarantined such that the overall rate of disease transmission in a given region is insensitive to the number of hospitalized persons in that region.

Estimates of 12 Fixed Parameter Values of the Compartmental Model

We summarize the rationale for each of our estimates for the values of the following 12 parameters of the compartmental model: m_b, ρ_E, ρ_A, k_L, k_Q, j_Q, f_A, f_H, c_A, c_I, f_R, and c_H. The estimates are assumed to apply to all regions (i.e., we take these parameters to have region-independent values). We provide rough provisional estimates below because we had limited input information for the estimates. Although using point estimates for some of the model parameters can lead to underestimates of parametric uncertainty *(9)*, aggressively leveraging prior knowledge (namely, parameter point estimates) reduces the number of adjustable parameters, which is necessary because not all model parameters can be inferred from case reporting. For each region of interest, we focused on inferring model parameters to characterize when disease transmission started (t_0), how disease transmission depends on behavior (σ, p_0, λ_0, and β), and surveillance (f_D and r). Given the data streams analyzed, the evident influence of behavior and social distancing on disease transmission, and our goal of situational awareness, focusing on inference of these parameters seems reasonable. As we discuss below, we fix the value of m_b, which characterizes social distancing, only because we found it correlated with the value of p_0, another social distancing parameter, when both are inferred.

The parameter m_b characterizes the effects of social distancing on disease transmission. Without social distancing, all contacts responsible for disease transmission are between mixing persons in the I_M and S_M pools and the rate of transmission is characterized by β. With social distancing, contacts can involve 1 person in a mixing population (I_M and S_M pools) and 1 person in a protected population (S_P and I_P pools); we characterized transmission rates associated by these contacts as $m_b\beta$ in the model. Contacts also can involve 2 persons in protected populations (I_P and S_P pools) and we characterize transmission associated with these types of contact by $m_b^2\beta$.In the model, the rates of transmission associated with these types of contacts are characterized by $m_b\beta$ and $m_b^2\beta$, respectively. We are confident that social distancing is protective (i.e., $m_b < 1$) but little information is available to suggest the magnitude of the effect. We arbitrarily set $m_b = 0.1$, which can be interpreted to mean that a susceptible person practicing social distancing has a 10-fold smaller chance of becoming infected than a susceptible person that is not practicing social distancing. In exploratory analyses, wherein we allowed m_b to be a free parameter, we found that its inferred value is positively correlated with the extent of social distancing, which is determined by the relevant social distancing setpoint parameter; for example, p_0 during the initial social-distancing period. Thus, we interpret the inferred quasistationary value of S_P to be an effective population size. If our estimate for m_b is too high (i.e., we underestimate the protective effect of social distancing), the effective size will be larger than the true size. Conversely, if our estimate for m_b is too low, the effective size will be smaller than the true size.

The parameter ρ_E characterizes the relative infectiousness of persons without symptoms during the incubation period; ρ_A characterizes the infectiousness of those without symptoms in the immune clearance phase. Infectiousness is compared to that of a symptomatic person. Using a 1-step real-time reverse transcriptase PCR (rRT-PCR) assay to quantify viral RNA abundance in nasopharyngeal and oropharyngeal samples, Arons et al. (*10*) determined rRT-PCR cycle threshold (C_t) values for 17 symptomatic and 24 presymptomatic persons. C_t value is inversely proportional to abundance. In the study, Arons et al. noted symptomatic persons had typical symptoms and asymptomatic persons lacked symptoms at the time of testing but developed symptoms $\leq$1 week after testing. At the time of testing, the median C_t value was 24.8 for symptomatic persons and 23.1 for presymptomatic persons. On the basis of these results and an

assumption that infectiousness is proportional to viral load, we estimated that $\rho_E = 1.1$. An estimate for ρ_E >1 is consistent with the findings of He et al. (*11*), who inferred that viral load peaks 0.7 days before the onset of symptoms from an analysis of temporal viral load data and information available about infector–infectee transmission pairs. A review of the literature by A. Benefield (unpub. data, https://www.medrxiv.org/content/10.1101/2020.09.28.20202028v1) indicates that viral load is maximal before onset of symptoms. Over a period of 19 days, Nguyen et al. (*12*) performed daily rRT-PCR assays for viral RNA in nasopharyngeal samples from 17 symptomatic and 13 asymptomatic persons. Ngyuen et al. (*12*) developed a curve-fitting model for each group to characterize their viral decay kinetics. These models indicate that the mean C_t for symptomatic persons was roughly 90% of the mean C_t for asymptomatic persons over the first week of the study, after which most persons tested negative or had a C_t near the threshold of detection, $C_t = 40$. Thus, we estimate that $\rho_A = 0.9$, but our estimates of ρ_E and ρ_A should be considered crude.

The parameter k_L characterizes the duration of the incubation period. In the model, the incubation period is divided into 5 stages (for reasons explained shortly). The waiting time for completion of all 5 stages is described by an Erlang distribution with a shape parameter $k = 5$ and a scale parameter $\mu = 1/k_L$. Lauer et al. (*1*) estimated times of exposure and symptom onset for 181 confirmed cases and found that the median time between SARS-CoV-2 infection and onset of COVID-19 symptoms is 5.1 days. Lauer et al. (*1*) also found that the empirical distribution of waiting times is fit by an Erlang distribution with $k = 6$ and $\mu = 0.88$ days, which suggests that the empirical waiting time distribution can be reproduced by dividing the incubation period into 6 stages and setting $k_L = 1.14$ d^{-1}. However, an Erlang distribution with $k = 5$ and $\mu = 1.06$ d has a nearly identical shape. Because simulation costs are reduced by dividing the incubation period into 5 instead of 6 stages, we considered 5 stages in the model. The distribution of waiting times estimated by Lauer et al. (*1*) is reproduced by our model when we set $k_L = 0.94$ d^{-1}.

The parameters k_Q and j_Q characterize testing driven quarantine and symptom driven self-isolation. We assume that testing is random. Thus, the number of infected persons moving into quarantine per day is the number of infected persons subject to quarantine times the fraction of the total population tested per day times a multiplier capturing the effect of contact tracing.

We take the multiplier to be average household size, 2.5 (*6*). Thus, assuming ≈500,000 tests per day in the United States (The COVID Tracking Project, https://covidtracking.com/data/us-daily) and a total population of 330 million (US Census Bureau, https://www.census.gov/popclock), we estimate $k_Q = 0.0038$ d^{-1}. The k_Q parameter, which characterizes the rate at which exposed persons move to quarantine because of testing and contact tracing, incorporates factors such as false negative test results. As a simplification, we assume that k_Q is the same for each stage of disease progression. We assume $j_Q = 0.4$ d^{-1}. With this setting, the median waiting time from onset of symptoms to initiation of self-isolation is approximately 40 hours. A faster timescale for self-isolation is probably not realistic despite general awareness of the COVID-19 pandemic, because as considered in the study of Böhmer et al. (*13*), for any given person, any give person can have a prodromal phase of ≈1 day marked phase of ≈1 day marked by non-COVID-19–specific symptoms other than fever and cough.

The parameter f_A is the fraction of infected persons who never develop symptoms. We estimate f_A on the basis of information about the COVID-19 outbreak on the *Diamond Princess* cruise ship, as recounted by Sakurai et al. (*14*) and others (*15*,*16*). Before disembarking, 3,618 passengers and crew members were tested for SARS-CoV-2 infection. Of the 712 persons testing positive for SARS-CoV-2, 410 were without symptoms at the time of testing. The Ministry of Health, Labour, and Welfare of Japan (*18*) reported that 311 (76%) of these persons remained asymptomatic over the course of long-term follow-up (*15*,*16*). Thus, we estimate that $f_A = \frac{311}{712} \approx 0.44$, which is consistent with the results of other studies. Lavezzo et al. (unpub. data, https://www.medrxiv.org/content/10.1101/2020.04.17.20053157v1) estimated that 43% of all infections are asymptomatic. Gudbjartsson et al. (*18*) found 7/13 persons detected to have SARS-CoV-2 infection in random-sample population screening did not report symptoms; 43% of all SARS-CoV-2–positive participants in the study were symptom-free.

The parameter f_H is the fraction of symptomatic persons progressing to severe disease. We set f_H such that our model predicts a uniform infection fatality rate (IFR) consistent with that determined by Perez-Saez et al. (*19*) from serologic survey results and death incidence reports, 0.0064 (≈0.64%). Others reported similar IFR estimates (R. Grewelle and G. De Leo, unpub data, https://www.medrxiv.org/content/10.1101/2020.05.11.20098780v1.full.pdf). According to our model, IFR is given by $(1 - f_A)f_H(1 - f_R)$, which is the fraction of all infected cases

predicted to have symptoms develop, then progress to severe disease and hospitalization, and finally a fatal outcome. Thus, based on our estimates for f_A (0.44) and f_R (0.79) and the empirical IFR (0.0064), we set $f_H = \frac{0.0064}{0.56 \times 0.21} \approx 0.054$.

The parameter c_A characterizes the duration of infectiousness of asymptomatic persons in the immune response phase. For each of 89 asymptomatic individuals, Sakurai et al. (*14*) reported the time between the first positive PCR test for SARS-CoV-2 and the first of 2 serial negative PCR tests. The mean duration of this period was ≈9.1 days. We assume that this period coincides with the period of infectiousness and that this period encompasses both the incubation period and the immune response phase. With the incubation period for both presymptomatic and asymptomatic persons divided into 5 stages of equal mean duration $1/k_L$, the overall mean duration of the incubation period is $5/k_L$. Based on our earlier estimate that $k_L = 0.94\ \mathrm{d}^{-1}$, the mean duration of the incubation period is estimated as 5.3 days. Accordingly, the mean duration of the immune clearance phase for asymptomatic persons is estimated as $9.1\ \mathrm{d} - 5.3\ \mathrm{d} = 3.8\ \mathrm{d}$, and it follows that $c_A = \frac{1}{3.8\ \mathrm{d}} \approx 0.26\ \mathrm{d}^{-1}$.

If $f_H \ll 1$, the parameter c_I characterizes the duration of infectiousness of persons who develop mild COVID-19 symptoms (i.e., symptoms not severe enough to require hospitalization). Wölfel et al. (*20*) attempted to isolate live virus from clinical throat swab and sputum samples collected from 9 patients at multiple time points after the onset of mild COVID-19 symptoms. Roughly 67% of attempts at 6 days, 38% at 8 days, and 0 at 10 days were successful at isolating virus. Assuming that a negative culture coincides with loss of infectiousness, we estimate that $c_I = -\frac{\ln(0.38)}{8\ \mathrm{d}} \approx 0.12\ \mathrm{d}^{-1}$.

The parameters f_R and c_H characterize the hospital stays of the severely ill. These parameters affect predictions of COVID-19–caused deaths and hospital resource utilization but do not affect the predicted transmission dynamics, because we assume that hospitalized patients are effectively quarantined and do not contribute significantly to disease transmission (i.e., there is no I_H term in ϕ_M or ϕ_P [equations 18 and 19]). The parameter f_R is the fraction of hospitalized patients who recover, and the parameter c_H characterizes the rate at which patients are discharged, either because they recovered or died. Richardson et al. (*21*) reported that the overall median length of hospital stay for 2,634 discharged patients (alive or dead) was 4.1 days.

Thus, we estimate that $c_H = \frac{\ln(2)}{4.1\text{ d}} \approx 0.17\text{ d}^{-1}$. Among the discharged patients, 553 (21%) died (*21*). Thus, we estimate that $f_R = 0.79$.

Likelihood Function Used in Inference of Model Parameter Values

We assumed that the likelihood of a set of adjustable parameter values θ_F given a report of δC_i new cases on calendar date $\mathcal{D}_i$, which we will denote as $\mathcal{L}_i(\theta_F; \delta C_i)$, is given by the following equation:

$$\mathcal{L}_i(\theta_F; \delta C_i) = \text{nbinom}(\delta C_i; r, p_i) = \binom{\delta C_i + r - 1}{\delta C_i - 1} p_i^r (1 - p_i)^{\delta C_i} \quad [25]$$

where δC_i is a nonnegative integer (the number of new cases reported), i is an integer indicating the date $\mathcal{D}_i$ or the period (t_i, t_{i+1}); $\text{nbinom}(\delta C_i; r, p_i)$ is the probability mass function of the negative binomial distribution $\text{NB}(r, p_i)$, which has 2 parameters, $r > 0$ and $p_i \in [0,1]$; and θ_F is a model-dependent ordered set of feasible (i.e., allowable) values for the adjustable model parameters (e.g., N, t_0, k, and θ in the case of the curve-fitting model) augmented with a feasible value for r. We defined t_i as t $t_i \equiv t_0 + i$ d, where $t_0 > 0$ is 00:00 hours of $\mathcal{D}_0$, the start date of the local epidemic. We take the dispersion parameter r of $\text{NB}(r, p_i)$ to be date and time-independent (i.e., applicable to all surveillance days) and infer the value of r jointly with the values of the model parameters.

As stated previously, we consider surveillance testing to be a stochastic process in that we assume that the fraction of cases detected varies stochastically from day to day. Furthermore, we assume that the randomness in the number of new cases detected on a given date t_i is captured by a negative binomial distribution $\text{NB}(r, p_i)$, where r is a ., t_i-independent parameter and p_i is a t_i-dependent parameter. Our assumptions mean that, for each date t_i, we are taking the predicted number of new cases, $I(t_i, t_{i+1})$, to correspond to $\mathbb{E}[\text{NB}(r, p_i)]$, which equals $r(1 - p_i)/p_i$. For this relationship to hold true, each distributional parameter p_i must satisfy the following constraint:

$$p_i = \frac{r}{r + I(t_i, t_{i+1})}. \quad [26]$$

We use this constraint to determine the value of p_i for all t_i. As stated previously, the value of r is jointly inferred with the values of the adjustable compartmental model parameters.

If $m + 1$ daily case reports are available, from date $\mathcal{D}_0$ to date $\mathcal{D}_m$, we assumed that each likelihood $\mathcal{L}_i(\theta_F; \delta C_i)$ given by equations 25 and 26 is independent. Thus, we have:

$$\mathcal{L}(\theta_F; \{\delta C_i\}_{i=0}^{m}) = \prod_{i=0}^{m} \log \mathcal{L}_i(\theta_F\,; \delta C_i) \qquad [27]$$

where $\mathcal{L}(\theta_F; \{\delta C_i\}_{i=0}^{m})$ is the likelihood of θ_F given all available case reports $\{\delta C_i\}_{i=0}^{m}$. As stated previously, δC_i is the number of new cases reported on date $\mathcal{D}_i$ and $I(t_i, t_{i+1})$ is the model prediction of δC_i. Furthermore, recall that θ_F is defined as a model-dependent ordered set of feasible adjustable model parameter values augmented with a feasible value for the likelihood function parameter r. The identity of θ_F depends on whether we are using equation 27 to make inferences conditioned on the curve-fitting model or the compartmental model, meaning we use equation 27 in both cases but the identity of θ_F depends on the model being considered. The ordering of parameter values within the set θ_F is arbitrary but should be consistent.

When we use equations 25–27 in combination with the compartmental model and select $n = 0$, the elements of θ_F are t_0, σ, p_0, λ_0, β, and f_D. In this case, we obtained $I(t_i, t_{i+1})$ from equation 24. When we use equations 25–27 in combination with the curve-fitting model, the elements of θ_F are N, t_0, k, θ, and r. In this case, we obtained $I\ [t_i, t_{i+1}]$) from equation 31.

Bayesian Inference and Online Learning

We chose the Bayesian inference framework to parametrize the models with uncertainty quantification. In Bayesian inference, given a set of data D, the probability of each set of the parameters, denoted in θ_F, is constrained by the Bayes formula:

$$\mathbb{P}\{\theta_F|D\} = \frac{\mathbb{P}\{D|\theta_F\}\ \mathbb{P}\{\theta_F\}}{\int_\Omega \mathbb{P}\{\theta_F{}'|D\}\ \mathbb{P}\{\theta_F{}'\}\ \mathrm{d}\theta_F{}'}\,. \qquad [28]$$

Here, $\mathbb{P}\{\theta_F\}$ is the prior parameter distribution, which represents our belief of how the model parameters should distribute in the parameter space Ω, and $\mathbb{P}\{D|\theta_F\}$ is the likelihood of the parameter set θ_F given the dataset D, that is, $\mathcal{L}(\theta_F; \{\delta C_i\}_{i=0}^{m})$ in equation 27. In general, evaluating the posterior parameter distribution $\mathbb{P}\{\theta_F|D\}$ is a difficult computation, mainly because of the high-dimensional integration of the term $\int_\Omega \mathbb{P}\{\theta_F{}'|D\}\ \mathbb{P}\{\theta_F{}'\}\ \mathrm{d}\theta_F{}'$, a term often

referred to as the evidence. Thus, for high-dimensional models, we relied on Markov chain Monte Carlo (MCMC) techniques to sample the posterior parameter distribution $\mathbb{P}\{\theta_F|D\}$.

In contrast of many modeling analyses that focus on identifying parameter distributions, we were interested in projections of models in which parameters are inferred by past data to project into the future. To this end, we evaluated our model with a probabilistic parameter set distributed by the obtained posterior distribution $\mathbb{P}\{\theta_F|D\}$. Formally, we denoted the prediction of the confirmed cases between future day t_i and t_{i+1} by our deterministic model with a set of parameters θ_F by $I(t_i, t_{i+1}; \theta_F)$. Our deterministic prediction represents the mean of the fundamentally random new confirmed cases reported in a future interval (t_i, t_{i+1}). If only parametric uncertainty that propagates through the deterministic model is available, the confirmed cases reported in a future interval (t_i, t_{i+1}) would be distributed according to $\int_\Omega I(t_i, t_{i+1}; \theta_F)\ \mathbb{P}\{\theta_F|D\}\ \mathrm{d}\theta_F$. However, observation noise also is apparent, for which we model by a negative binomial distribution. Observation noise also needs to be injected into the prediction to quantify the full uncertainty. The full prediction, accounting for parametric uncertainty, is a random variable distributed according to:

$$\int_\Omega \mathrm{nbiom}\left(i; r, \frac{r}{r + I(t_i, t_{i+1}; \theta_F)}\right)\ \mathbb{P}\{\theta_F|D\}\ \mathrm{d}\theta_F. \quad [29]$$

In practice, the above random variable is resampled from the posterior chain derived from the MCMC sampling. We denoted the MCMC posterior chain by $\left\{\theta_F^{(1)}, \theta_F^{(2)} \dots \theta_F^{(N)}\right\}$. We sampled the posterior chain and denoted the resampled parameter set by θ_F^s and the deterministic prediction of that resampled parameter in interval (t_i, t_{i+1}) by $I(t_i, t_{i+1}; \theta_F^s)$. Then, we generated a negative binomial random number in which we set the first parameter of the negative binomial distribution as $r_s/(r_s + I(t_{i+1}, t_i; \theta_F^s))$ where r_s is the resampled r and also is a free parameter in θ_F^s and is inferred in the MCMC. We repeated the resampling procedures and used the generated samples to compute the percentile of the historic observations and future predictions.

Our aim was to perform the Bayesian inference daily as soon as a new regional confirmed case number was reported. Although the Bayesian framework enabled a sequential analysis, we used the previous derived posterior distribution as a prior so that new inference problems involved only one new data point. In practice, such an analysis is difficult if the posterior distribution cannot be emulated or interpolated from the discrete posterior chain. Our

analysis showed that in some regions, the posterior was far from Gaussian, making accurate interpolation or emulation difficult. Thus, we did not adopt this workflow, and instead, we conducted the inference with all the data points collected up to the time of inference. Nevertheless, we started the MCMC chain from the maximum a posteriori estimator estimated from the previous derived chain, and we also used the previously derived chain for estimating the optimal covariance matrix for the proposal of the normal symmetric random-walk Metropolis sampler. Our approach enabled online learning of the optimal proposal, which significantly reduces the mixing time.

Technical Details of Approach and Numerical Methods Used in Bayesian Inference

Because the variability of the data due to the regional and temporal differences, it is difficult to identify a universal sampling strategy (the proposal kernel). Thus, we adopted an adaptive Metropolis algorithm, specifically Algorithm 4 in Andrieu and Thoms (*22*) to accommodate the regional and temporal differences.

For all the model parameters, we assume their priors are uniformly distributed. Denote the 0:00 of the calendar date of the first confirmed case in a specific region by t_{first} and the total population of that region by S_0. For the curve-fitting model, we assume the parameters are bounded by $N \in (0, S_0)$, $t_0 \in (t_{\text{first}} - 21, t_{\text{first}})$, $\mu \in (0,10^6)$, $k \in (0,10^6)$, $\theta \in (0,10^6)$. For the compartmental model with $n = 0$, we assume that the parameters are bounded by $t_0 \in (0, t^\star)$, $\sigma \in (t_0, t^\star - t_0)$, $\beta \in (0,10^6)$, $\lambda_0 \in (0,10)$, $p_0 \in (0,1)$, and $f_D \in (0,1)$, where $t^\star$ is the time at which inference is performed. We assume $r \in (0,10^6)$ for both models. The limits on parameter values reflect feasibility constraints or bounds determined by trial-and-error to ensure that the posterior mass is contained within the hypercube defined by the limits. We adopted rejection-based sampling to assure the parameter values are sampled in the hypercube.

We start inference with an isotropic proposal kernel that randomly perturbs the parameter values by independent Gaussian proposals for which standard deviations are set to be 5% of the parameter values. We carry out the standard Metropolis–Hastings algorithm for 5×10^4 iterations first to identify local minimum. Then, we turned on the adaptive Metropolis algorithm to calculate the covariance matrix on-the-fly for another 5×10^4 iterations, after which we

turned on an on-the-fly learning for optimal proposed increment by using λ from algorithm 4 of Andrieu and Thoms (*22*). Because the weight for learning the proposed increment decays $1/\mathrm{iteration}$ (*22*), the proposed increment stabilizes after about 10^3 more iterations. We began to collect the statistics from 1.5×10^5 iteration until the simulation finishes at 6×10^5 iterations.

Except for the first time of the procedure (i.e., online learning and day-to-day operation), we warm-started the simulation from the previously obtained best-fit (maximum a posteriori estimator) and with the previously obtained covariance matrix and proposed increment. We carried out standard Metropolis–Hastings algorithms for 2.5×10^4 iterations, first to identify a local minimum noting that it was often not far from the previously-identified maximum a posteriori estimate. Then, we turned on the adaptive MCMC algorithm to calculate the covariance matrix on-the-fly for another 5×10^4 iteration. Then we used another 2.5×10^4 iterations to calculate the optimal proposed increment. We started to collect statistical information from 1×10^5 to 4×10^5 iterations when the simulation finishes.

Model Selection

We used a heuristic model-selection procedure to select the most parsimonious value of n, which determines the structure of the compartmental model (i.e., the number of social distancing periods considered) and the number of adjustable parameters describing social distancing (3 for each social-distancing period). The procedure for deciding between $n = 0$ and $n = 1$ is as follows. We calculated Aikake information criterion (AIC) (*23*) values for $n = 0$ and $n = 1$ versions of the compartmental model. Similarly, we calculated Bayesian information criterion (BIC) (*23*) values for the 2 versions of the model. We defined ΔAIC as $\mathrm{AIC}^{n=0} - \mathrm{AIC}^{n=1}$ and ΔBIC as $\mathrm{BIC}^{n=0} - \mathrm{BIC}^{n=1}$, where $\mathrm{AIC}^{n=0}$ ($\mathrm{AIC}^{n=1}$) is the AIC value calculated for the $n = 0$ ($n = 1$) version of the compartmental model and, similarly, $\mathrm{BIC}^{n=0}$ ($\mathrm{BIC}^{n=1}$) is the BIC value calculated for the $n = 0$ ($n = 1$) version of the compartmental model. Burnham and Anderson (*23*) suggest we can interpret ΔAIC>10 to mean that the evidence strongly supports the $n = 1$ version of the model. In other words, we are justified in using $n = 1$ (the more complex version of the model) instead of $n = 0$ when ΔAIC>10. However, we take a more conservative approach. We adopt $n = 1$ over $n = 0$ only when both ΔAIC>10 and ΔBIC>10. We are using the same approach described above to decide between $n = 1$ and $n = 2$.

Prediction Updates

Daily predictions based on region-specific parameterizations of the compartmental model are being archived at a GitHub repository (https://github.com/lanl/COVID-19-Predictions). It should be noted that the model's structural parameter n, which determines the number of distinct social-distancing periods considered in the model, varies from region to region and can change over time.

Forecasting Accuracy

We performed out-of-sample testing of forecasting accuracy for inferences conditioned on the compartmental model and chose value of n through model selection. For predictions 1, 4, and 7 days ahead, we determined the empirical coverage, meaning the frequencies characterizing how often the empirical data fell below predicted quantiles. In this analysis, we considered surveillance data available during July 14–September 9, 2020. We obtained results using the combined out-of-sample data and predictions for all 15 metropolitan statistical areas (MSAs) in our study (Appendix Figure 5). As shown, our overall predictions are biased toward underprediction of new case detection, although the bias is weak.

Evaluation of a Stochastic Version of the Compartmental Model

A compartmental model may be formulated as either a deterministic model or a stochastic model. Use of a deterministic model is a potential liability, because assumptions of a deterministic model can break down in some situations, necessitating use of a stochastic model. For this reason, we performed an analysis to determine whether a deterministic or stochastic would be better suited for analysis of our data of interest.

We constructed a person-based continuous-time Markov chain with consideration of the same processes as those in our deterministic compartmental model. The intrinsic stochasticity arises from the discreteness of populations. We followed the standard procedure to construct a set of stochastic differential equations (SDEs), by first formulating a master equation, then performing the Kramers-Moyal expansion to obtain the approximate continuum-limit Fokker-Planck equation in the large-population limit, and then formulating the corresponding SDEs by using the procedure described by Lin et al. (*24*,*25*).

In simulations, we used the Euler-Maruyama integrator to evolve the SDEs with a time step of 0.05 days. We ensured the timestep was sufficiently small through a trial-and-error approach.

We adopted a standard particle filter technique to identify the Maximum Likelihood Estimator of the parameters of the stochastic model, noting that our process is not time-homogeneous due to different episodes of distinct social distancing practices. We used the data from the New York City (New York) and Miami (Florida) MSAs from January 21–August 29, 2020 to parametrize the stochastic model.

After identifying the point estimator, we calculated the predicted quantiles as for the deterministic model. Finally, we quantified the in-sample empirical coverage (i.e., the empirical frequencies quantifying when training-data points fall below the predicted quantiles). When the noise model is correct, one would expect that such frequencies should coincide with the prediction.

For the New York City MSA, we successfully reproduced the case reporting data (Appendix Figure 6). For the Miami MSA, we also successfully reproduced the case reporting data (Appendix Figure 7). We note that the New York City epidemic curve has a single peak, whereas the Miami epidemic curve has 2 peaks. By visually inspecting the predictive posteriors, we noted that the stochastic models lead to higher uncertainty than the deterministic models for New York, NY and Miami, FL (Appendix Figures 6,7). The in-sample calibration curves show that the SDE model in both cases is farther away from the diagonal than the deterministic model, indicating that the stochastic models assert more uncertainty than the data distribution (Appendix Figure 6, panel D; Appendix Figure 7, panel D). Thus, deterministic versions of the model seem more appropriate for forecasting than stochastic versions, at least currently.

Evaluation of a Curve-Fitting Model

Various fitting functions, or curve-fitting models, have been used to reproduce COVID-19 incidence data and to make forecasts of new cases (*26–31*; IHME COVID-19 Health Service Utilization Forecasting Team, unpub. data, https://www.medrxiv.org/content/10.1101/2020.03.27.20043752v1; Nishimoto Y, unpub. data, https://www.medrxiv.org/content/10.1101/2020.07.02.20144899v2). We decided to evaluate this

approach by using the fitting function described below, which is a discretized convolution of 2 integrals. This approach is related to an approach used to analyze AIDS data (*32*,*33*). Use of a curve-fitting model has the advantage of not requiring insights into disease transmission mechanisms, which can be particularly advantageous during outbreaks of new or emerging diseases. A drawback of a curve-fitting model is that it might be limited in its ability to reproduce empirical epidemic curves. We considered a fitting function that can generate an asymmetric curve, such as an epidemic curve with 2 timescales, in which fast growth and slow decay in new daily cases occur (Appendix Figure 8). Like many such models used in epidemiology, the curve-fitting model considered here can generate only single-peaked curves. Because the MSAs of interest have experienced multiple-wave disease-transmission dynamics (i.e., >1 period of increasing disease incidence), we are no longer using the curve-fitting model in forecasting. We present the curve-fitting model and results obtained with it to illustrate how a curve-fitting model can be combined with Bayesian inference to generate forecasts with uncertainty quantification.

For each MSA of interest, we assumed that there is an infection curve $Q(t)$ describing the number of persons who become infected at time t with SARS-CoV-2 and who will later be detected in local COVID-19 surveillance efforts. Furthermore, we assumed that this curve has a shape that can be generated and reproduced by $\rho_\Gamma(t, k, \theta)$, the probability density function of a gamma distribution $\Gamma(k, \theta)$. In other words, we assumed that $Q(t) = N\rho_\Gamma(t, k, \theta)$, where N is a scaling factor that we can identify as the number of persons who will be detected over the entire course of the local epidemic. The shape of a gamma distribution is flexible and determined by the values of its two parameters: k, which is called the shape parameter, and θ, which is called the scale parameter. The functional form that we assume for $Q(t)$ allows the curve-fitting model to reproduce the shape of an epidemic curve having 2 timescales. Early in the pandemic, many empirical COVID-19 epidemic curves appeared to have 2 timescales: an initial period during which new case reports increase relatively quickly from day to day followed by a period during which new case reports decrease relatively slowly from day to day.

We did not take the infection curve $Q(t)$ to correspond directly to the number of new COVID-19 cases reported on the date encompassing time t, because only symptomatic persons are likely to be detected in COVID-19 surveillance testing (to a first approximation). This

situation complicates our model because a potentially lengthy, variable delay in the onset of symptoms after infection is known (*1*). We assume that the waiting time $\tau - t$ for the onset of COVID-19 symptoms after SARS-CoV-2 infection at time t is distributed according to a log-normal distribution. Let us use $\rho_{LN}(\tau - t; \mu_{LN}, \sigma_{LN})$ to denote the probability density function of the waiting-time distribution modeled by a log-normal distribution with parameters μ_{LN} and σ_{LN} set to the values estimated by Lauer et al. (*1*). We used $I(t_i, t_{i+1})$ to denote the predicted number of new COVID-19 cases reported within a period beginning at time $t_i \equiv t_0 + i$ d and ending at time t_{i+1}, where $t_0 > 0$ is the start time of the local epidemic. We assumed that surveillance testing for SARS-CoV-2 infection starts prior to time t_0, and we took time $t = 0$ to correspond to 00:00 hours on January 21, 2020, the date on which detection of the first US COVID-19 case was widely reported (*34*). Under these assumptions, $I(t_i, t_{i+1})$ is given by a convolution of integral functions by the following expression:

$$I(t_i, t_{i+1}) = N \int_{t_i}^{t_{i+1}} \int_{t_0}^{\tau} \rho_{LN}(\tau - s; \mu_{LN}, \sigma_{LN})\, \rho_{\Gamma}(s - t_0; k, \theta)\, \mathrm{d}s\, \mathrm{d}\tau \qquad [30]$$

Of note, s in this equation is a dummy variable of integration. Equation 30 is a special case of the general model proposed by Brookmeyer and Gail (*32*) for predicting future AIDS cases.

Equation 30 can be evaluated through numerical quadrature, but this procedure is computationally expensive. To overcome this limitation, we replaced the double integral in equation 30 with a sum, and we calculate $I(t_i, t_{i+1})$ using the following expression instead:

$$I(t_i, t_{i+1}) = K_0 Q_i + K_1 Q_{i-1} + \cdots + K_{i-1} Q_1 + K_i Q_0 = \sum_{j=0}^{i} K_{i-j} Q_j \qquad [31]$$

where

$$K_{i-j} = \int_{t_{i-j}}^{t_{i-j+1}} \rho_{LN}(t; \mu_{LN}, \sigma_{LN}) dt = F_{LN}\big(t_{i-j+1}; \mu_{LN}, \sigma_{LN}\big) - F_{LN}(t_{i-j}; \mu_{LN}, \sigma_{LN}) \qquad [32]$$

and

$$Q_j = N \int_{t_j}^{t_{j+1}} \rho_{\Gamma}(t - t_0; k, \theta) dt = N[F_{\Gamma}\big(t_{j+1} - t_0; k, \theta\big) - F_{\Gamma}\big(t_j - t_0; k, \theta\big)] \qquad [33]$$

In equation 31, the K_{i-j} terms are weighting functions (i.e., kernels) that account for the variable duration of the incubation period, and the Q_j terms represent cumulative numbers of new detectable infections occurring over discrete 1-day periods. In equation 32, each F_{LN} term denotes a cumulative distribution function (CDF) of a log-normal distribution, and in equation 33, each F_Γ term denotes a cumulative distribution function of a gamma distribution. In other words, Q_j is the cumulative number of persons infected in the period (t_j, t_{j+1}) who will eventually be detected, and K_{i-j} is the probability that one of these persons becomes symptomatic and is detected in the period (t_i, t_{i+1}), where $t_i \geq t_j$.

The functional form of our curve-fitting model is defined by equations 31–33, which are derived from equation 30. As can be seen by inspecting equation 30, the curve-fitting model has 6 parameters: N, t_0 (which is hidden in the definition of t_i), k, θ, μ_{LN}, and σ_{LN}. As we noted previously, estimates are available for μ_{LN} and σ_{LN} from Lauer et al. (*1*). These parameters characterize the variable duration of the incubation period, which starts at infection and ends at the onset of symptoms. Thus, we take μ_{LN} and σ_{LN} to have fixed region-independent values. We take the remaining parameters, N (a population size/scaling factor), t_0 (the start time of the local epidemic), and k and θ (the parameters that determine the shape of the infection curve $Q(t)$), to have adjustable region-specific values. In our daily inferences, we considered 1 additional region-specific adjustable parameter, the dispersal parameter of the likelihood function (see equation 27 above). The value of this parameter, r, is inferred jointly with the values of N, t_0, k, and θ.

For the period of January–June 2020, for each of the 15 MSAs of interest, we parameterized on a daily basis a curve-fitting model for consistency with all daily reports of new confirmed cases available at the time. The methodology used was the same as that used for inferences conditioned on the compartmental model unless otherwise noted. For each MSA, the curve-fitting model was taken to have 4 adjustable parameters: N, the total number of infected persons who will be detected over the entire course of the local epidemic; t_0, the start time of the local epidemic; and k and θ, the shape and scale parameters of a gamma (Γ) distribution (Appendix Table 2). Inference of adjustable parameter values was based on a negative binomial likelihood function, which is given by equation 27. The dispersal parameter r of the likelihood was taken to be adjustable; its value was jointly inferred with those of N, t_0, k, and θ. Inferences

are conditioned on fixed parameter estimates for μ_{LN} and σ_{LN} (Appendix Table 2). These parameters, the mean and standard deviation of a log-normal distribution, characterized the incubation period (i.e., the waiting time from infection to onset of symptoms) (*1*). For any given (1-day) surveillance period and specified settings for parameter values, a prediction of the curve-fitting model for expected new cases detected was generated by evaluating the sum in equation 31. A prediction of the actual number of new cases detected was obtained by entering the predicted expected number of new cases (according to either the curve-fitting or compartmental model) into equation 29.

We obtained predictive inferences conditioned on the curve-fitting model for all 15 MSAs of interest (Appendix Figure 9). These results demonstrate that, for the timeframe of interest, the curve-fitting model was capable of reproducing many of the MSA-specific empirical epidemic curves. The limitations of curve fitting can be seen by examining predictions for the Atlanta, GA MSA, where we noted a high variability in the daily number of new cases detected. Although we did not see a clear downward trend in the data, the curve-fitting model nevertheless predicts a peak in late-April/early-May 2020 and a downward trend thereafter. This prediction is obtained because the model, by design, is only capable of generating single-peaked epidemic curves that rise and then fall.

References

1. Lauer SA, Grantz KH, Bi Q, Jones FK, Zheng Q, Meredith HR, et al. The incubation period of coronavirus disease 2019 (COVID-19) from publicly reported confirmed cases: estimation and application. Ann Intern Med. 2020;172:577–82. https://doi.org/10.7326/M20-0504 PubMed

2. Sia SF, Yan LM, Chin AWH, Fung K, Choy KT, Wong AYL, et al. Pathogenesis and transmission of SARS-CoV-2 in golden hamsters. Nature. 2020;583:834–8. PubMed https://doi.org/10.1038/s41586-020-2342-5

3. Kucirka LM, Lauer SA, Laeyendecker O, Boon D, Lessler J. Variation in false-negative rate of reverse transcriptase polymerase chain reaction-based SARS-CoV-2 tests by time since exposure. Ann Intern Med. 2020;173:262–7. PubMed https://doi.org/10.7326/M20-1495

4. Sekine T, Perez-Potti A, Rivera-Ballesteros O, Strålin K, Gorin JB, Olsson A, et al.; Karolinska COVID-19 Study Group. Robust T cell immunity in convalescent individuals with asymptomatic

or mild COVID-19. Cell. 2020;183:158–168.e14. PubMed https://doi.org/10.1016/j.cell.2020.08.017

5. Addetia A, Crawford KHD, Dingens A, Zhu H, Roychoudhury P, Huang ML, et al. Neutralizing antibodies correlate with protection from SARS-CoV-2 in humans during a fishery vessel outbreak with high attack rate. J Clin Microbiol. 2020;58:e02107-20. PubMed https://doi.org/10.1128/JCM.02107-20

6. To KKW, Hung IFN, Ip JD, Chu AWH, Chan WM, Tam AR, et al. COVID-19 re-infection by a phylogenetically distinct SARS-coronavirus-2 strain confirmed by whole genome sequencing. Clin Infect Dis. 2020 August 25 [Epub ahead of print]. PubMed https://doi.org/10.1093/cid/ciaa1275

7. United States Census Bureau. Metropolitan and micropolitan statistical areas population totals and components of change: 2010–2019. 2020 [cited 2020 Jul 1]. https://www.census.gov/data/tables/time-series/demo/popest/2010s-total-metro-and-micro-statistical-areas.html

8. Executive Office of the President. OMB bulletin no. 15-01. 2020 [cited 2020 Jul 1]. https://www.bls.gov/bls/omb-bulletin-15-01-revised-delineations-of-metropolitan-statistical-areas.pdf

9. Elderd BD, Dukic VM, Dwyer G. Uncertainty in predictions of disease spread and public health responses to bioterrorism and emerging diseases. Proc Natl Acad Sci U S A. 2006;103:15693–7. PubMed https://doi.org/10.1073/pnas.0600816103

10. Arons MM, Hatfield KM, Reddy SC, Kimball A, James A, Jacobs JR, et al.; Public Health–Seattle and King County; CDC COVID-19 Investigation Team. Presymptomatic SARS-CoV-2 infections and transmission in a skilled nursing facility. N Engl J Med. 2020;382:2081–90. https://doi.org/10.1056/NEJMoa2008457

11. He X, Lau EHY, Wu P, Deng X, Wang J, Hao X, et al. Temporal dynamics in viral shedding and transmissibility of COVID-19. [Erratum in: Nat Med. 2020;26:1491–3]. Nat Med. 2020;26:672–5. PubMed https://doi.org/10.1038/s41591-020-0869-5

12. Nguyen VVC, Vo TL, Nguyen TD, Lam MY, Ngo NQM, Le MH, et al. The natural history and transmission potential of asymptomatic SARS-CoV-2 infection. Clin Infect Dis 2020;ciaa711 https://doi.org/10.1093/cid/ciaa711

13. Böhmer MM, Buchholz U, Corman VM, Hoch M, Katz K, Marosevic DV, et al. Investigation of a COVID-19 outbreak in Germany resulting from a single travel-associated primary case: a case series. Lancet Infect Dis. 2020;20:920–8. PubMed https://doi.org/10.1016/S1473-3099(20)30314-5

14. Sakurai A, Sasaki T, Kato S, Hayashi M, Tsuzuki SI, Ishihara T, et al. Natural history of asymptomatic SARS-CoV-2 infection. N Engl J Med. 2020;383:885–6. https://doi.org/10.1056/NEJMc2013020 PubMed

15. Emery JC, Russell TW, Liu Y, Hellewell J, Pearson CAB, Atkins KE, et al.; CMMID COVID-19 Working Group. The contribution of asymptomatic SARS-CoV-2 infections to transmission on the Diamond Princess cruise ship. eLife. 2020;9:e58699. PubMed https://doi.org/10.7554/eLife.58699

16. Expert Taskforce for the COVID-19 Cruise Ship Outbreak. Epidemiology of the COVID-19 outbreak on cruise ship quarantined at Yokohama, Japan, February 2020. Emerg Infect Dis. 2020;26:2591–7. PubMed https://doi.org/10.3201/eid2611.201165

17. Ministry of Health, Labour and Welfare of Japan. Official report on the cruise ship *Diamond Princess*, May 1, 2020. 2020 [cited 2020 Jul 1]. https://www.mhlw.go.jp/stf/newpage_11146.html

18. Gudbjartsson DF, Helgason A, Jonsson H, Magnusson OT, Melsted P, Norddahl GL, et al. Spread of SARS-CoV-2 in the Icelandic population. N Engl J Med. 2020;382:2302–15. PubMed https://doi.org/10.1056/NEJMoa2006100

19. Perez-Saez J, Lauer SA, Kaiser L, Regard S, Delaporte E, Guessous I, et al. Serology-informed estimates of SARS-COV-2 infection fatality risk in Geneva, Switzerland. Lancet Infect Dis. 2020 Jul 14 [Epub ahead of print].

20. Wölfel R, Corman VM, Guggemos W, Seilmaier M, Zange S, Müller MA, et al. Virological assessment of hospitalized patients with COVID-2019. Nature. 2020;581:465–9. https://doi.org/10.1038/s41586-020-2196-x

21. Richardson S, Hirsch JS, Narasimhan M, Crawford JM, McGinn T, Davidson KW, et al.; the Northwell COVID-19 Research Consortium. Presenting characteristics, comorbidities, and outcomes among 5700 patients hospitalized with COVID-19 in the New York City area. JAMA. 2020;323:2052–9. https://doi.org/10.1001/jama.2020.6775

22. Andrieu C, Thoms J. A tutorial on adaptive MCMC. Stat Comput. 2008;18:343–73. https://doi.org/10.1007/s11222-008-9110-y

23. Burnham KP, Anderson DR. Multimodal inference: understanding AIC and BIC in model selection. Sociol Methods Res. 2004;33:261–304. https://doi.org/10.1177/0049124104268644

24. Lin YT, Kim H, Doering CR. Demographic stochasticity and evolution of dispersion I. Spatially homogeneous environments. J Math Biol. 2015;70:647–78. PubMed https://doi.org/10.1007/s00285-014-0776-9

25. Lin YT, Feng S, Hlavacek WS. Scaling methods for accelerating kinetic Monte Carlo simulations of chemical reaction networks. J Chem Phys. 2019;150:244101. PubMed https://doi.org/10.1063/1.5096774

26. Chen DG, Chen X, Chen JK. Reconstructing and forecasting the COVID-19 epidemic in the United States using a 5-parameter logistic growth model. Glob Health Res Policy. 2020;5:25. PubMed https://doi.org/10.1186/s41256-020-00152-5

27. Moreau VH. Forecast predictions for the COVID-19 pandemic in Brazil by statistical modeling using the Weibull distribution for daily new cases and deaths. Braz J Microbiol. 2020;51:1109–15. PubMed https://doi.org/10.1007/s42770-020-00331-z

28. Roosa K, Lee Y, Luo R, Kirpich A, Rothenberg R, Hyman JM, et al. Short-term forecasts of the COVID-19 epidemic in Guangdong and Zhejiang, China: February 13–23, 2020. J Clin Med. 2020;9:596. PubMed https://doi.org/10.3390/jcm9020596

29. Rypdal K, Rypdal M. A parsimonious description and cross-country analysis of COVID-19 epidemic curves. Int J Environ Res Public Health. 2020;17:E6487. PubMed https://doi.org/10.3390/ijerph17186487

30. Wu K, Darcet D, Wang Q, Sornette D. Generalized logistic growth modeling of the COVID-19 outbreak: comparing the dynamics in the 29 provinces in China and in the rest of the world. Nonlinear Dyn. 2020;101:1–21. PubMed https://doi.org/10.1007/s11071-020-05862-6

31. Xu J, Cheng Y, Yuan X, Li WV, Zhang L. Trends and prediction in daily incidence of novel coronavirus infection in China, Hubei Province and Wuhan City: an application of Farr's law. Am J Transl Res. 2020;12:1355–61. PubMed https://doi.org/10.1101/2020.02.19.20025148

32. Brookmeyer R, Gail MH. Minimum size of the acquired immunodeficiency syndrome (AIDS) epidemic in the United States. Lancet. 1986;2:1320–2. PubMed https://doi.org/10.1016/S0140-6736(86)91444-3

33. Brookmeyer R. Reconstruction and future trends of the AIDS epidemic in the United States. Science. 1991;253:37–42. PubMed https://doi.org/10.1126/science.2063206

34. Holshue ML, DeBolt C, Lindquist S, Lofy KH, Wiesman J, Bruce H, et al.; Washington State 2019-nCoV Case Investigation Team. First case of 2019 novel coronavirus in the United States. N Engl J Med. 2020;382:929–36. https://doi.org/10.1056/NEJMoa2001191

35. The New York Times. Coronavirus (Covid-19) data in the United States. 2020 [cited 2020 Jul 1]. https://github.com/nytimes/covid-19-data

Appendix Table 1. Strength-of-evidence comparison of compartmental models for daily new case counts of coronavirus disease in various metropolitan statistical areas, United States, January 21–June 26, 2020

MSA	ΔAIC*	ΔBIC*	$p_0^{n=0}$ (95% CI)†	$p_0^{n=1}$ (95% CI)†	$p_1^{n=1}$ (95% CI)†
New York City, NY	17	8.6	0.88 (0.85–0.90)	0.87 (0.80–0.89)	0.36 (0.11–0.83)
Los Angeles, CA	−6.5	−15	0.45 (0.38–0.45)	0.47 (0.42–0.80)	0.38 (0.33–0.97)
Chicago, IL	18	9.5	0.57 (0.46–0.61)	0.52 (0.46–0.75)	0.25 (0.03–0.68)
Dallas, TX	18	9.4	0.52 (0.41–0.52)	0.59 (0.49–0.77)	0.41 (0.33–0.60)
Houston, TX	50	42	0.39 (0.34–0.45)	0.49 (0.39–0.79)	0.30 (0.20–0.56)
Washington, DC	1.0	−7.5	0.39 (0.30–0.47)	0.77 (0.71–0.80)	0.68 (0.63–0.76)
Miami, FL	75	67	0.51 (0.46–0.57)	0.92 (0.81–0.97)	0.69 (0.61–0.80)
Philadelphia, PA	12	3.7	0.65 (0.57–0.69)	0.55 (0.49–0.81)	0.22 (0.03–0.69)
Atlanta, GA	9.9	1.5	0.54 (0.41–0.52)	0.58 (0.44–0.78)	0.29 (0.06–0.63)
Phoenix, AZ	66	58	0.43 (0.37–0.49)	0.55 (0.43–0.73)	0.34 (0.26–0.54)
Boston, MA	−31	−39	0.36 (0.29–0.37)	0.80 (0.69–0.85)	0.18 (0.06–0.97)
San Francisco, CA	20	12	0.32 (0.29–0.35)	0.36 (0.34–0.74)	0.17 (0.07–0.63)
Riverside, CA	3.8	−4.7	0.41 (0.36–0.46)	0.43 (0.38–0.74)	0.34 (0.03–0.48)
Detroit, MI	5.9	−2.6	0.75 (0.60–0.78)	0.80 (0.64–0.92)	0.93 (0.14–0.97)
Seattle, WA	55	46	0.87 (0.75–0.90)	0.82 (0.76–0.85)	0.59 (0.48–0.68)

*ΔAIC ≡ AIC$^{n=0}$ – AIC$^{n=1}$ and ΔBIC ≡ BIC$^{n=0}$ – BIC$^{n=1}$, where AIC$^{n=0}$ and AIC$^{n=1}$ are the AIC values calculated for the $n = 0$ and $n = 1$ versions of the compartmental model; BIC$^{n=0}$ and BIC$^{n=1}$ are the BIC values calculated for the $n = 0$ and $n = 1$ versions of the compartmental model (S. Anderson, unpub. data, https://www.medrxiv.org/content/10.1101/2020.04.17.20070086v1). CI, credible interval; AIC, Akaike information criterion; BIC, Bayesian information criterion.
†The first number in each entry in this column is the maximum a posteriori estimate.

Appendix Table 2. Parameters of the curve-fitting model and the associated-likelihood function used in predictive inference for daily new cases of coronavirus disease in the New York, NY metropolitan statistical area, January 21–June 21, 2020

Parameter	Estimate	Definition
N	470,000†	Population size
t_0	35 d†	Start of COVID-19 transmission
k	6.6†	Shape parameter of $\Gamma(k, \theta)$
θ	7.9†	Scale parameter of $\Gamma(k, \theta)$
μ_{LN}	1.6‡	μ-parameter of log-normal distribution
σ_{LN}	0.42‡	σ-parameter of log-normal distribution
p	Constrained§	Probability parameter of NB(r, p)
r	4.4†	Dispersal parameter of NB(r, p)

*N, t_0, k, θ, μ_{LN}, and σ_{LN} are parameters of the curve-fitting model; p and r are parameters of the associated-likelihood function.
†Estimates of the adjustable parameters (N, t_0, k, θ, and r) are region-specific and inference-time-dependent. Estimations made using data from the GitHub repository maintained by *The New York Times* newspaper (*35*). Time $t = 0$ corresponds to midnight on January 21, 2020.
‡Estimates of the fixed parameters μ_{LN} and σ_{LN} are those of Lauer et al. (*1*). These parameter estimates define a log-normal distribution that reproduces the empirical distribution of waiting times for the onset of symptoms after infection with severe acute respiratory syndrome coronavirus 2.
§The value of p is constrained (i.e., its reporting-time-dependent value is determined by a formula [Appendix equation 26]).

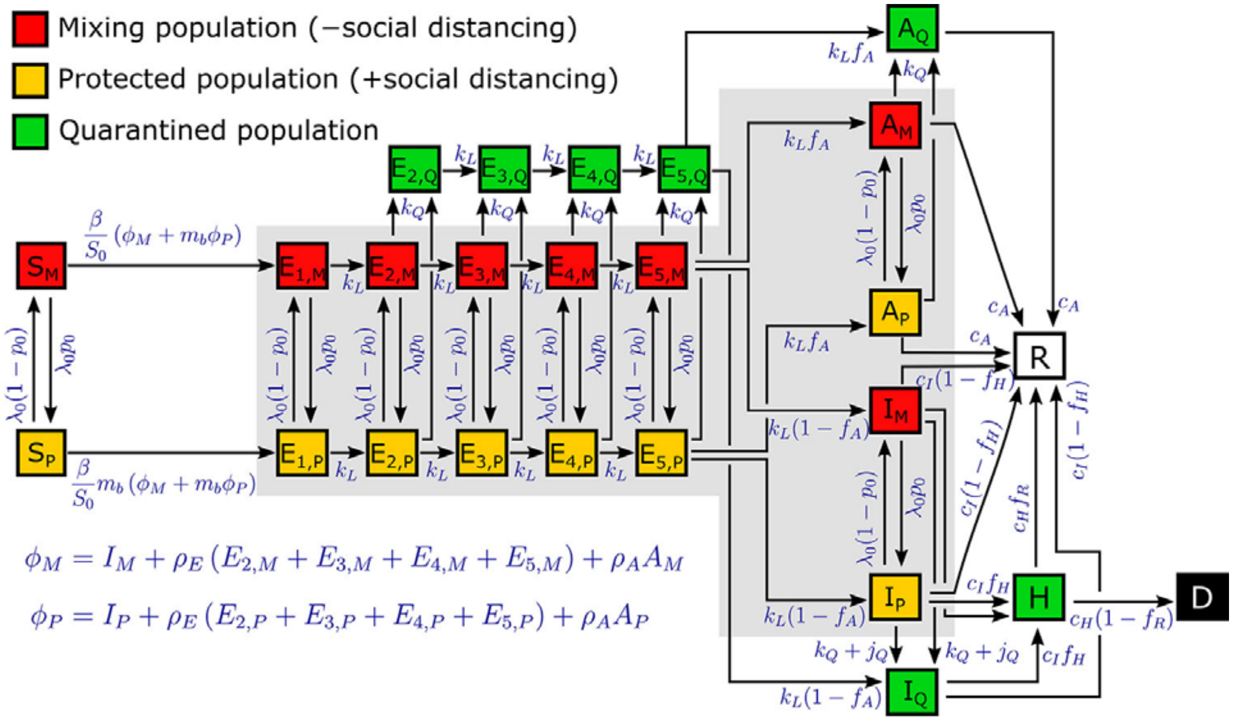

Appendix Figure 1. Detailed diagram of the populations and processes considered in the mechanistic compartmental model for daily new coronavirus disease cases during regional epidemics, United States, 2020. The model accounts for susceptible persons (S), exposed persons without symptoms in the incubation phase of disease (E), asymptomatic persons in the immune clearance phase of disease (A), mildly ill symptomatic persons (I), severely ill persons in hospital or at home (H), recovered persons (R), and deceased persons (D). The model also accounts for social distancing through mixing (i.e., not practicing social distancing) denoted by M and protected (i.e., practicing social distancing) subpopulations denoted by P subscripts; quarantined subpopulations denoted by a Q subscript; and self-isolation spurred by symptom awareness. We considered persons who are self-isolating because of symptoms to be members of the I_Q population. The incubation period is divided into 5 stages (E_1–E_5), which enables the model to reproduce an empirically determined (nonexponential) Erlang distribution of waiting times for the onset of symptoms after infection (*1*). The exposed population comprises presymptomatic and asymptomatic persons. The A populations consist of asymptomatic persons in the immune clearance phase. The gray background indicates the populations that contribute to disease transmission. An auxiliary measurement model (equations 23 and 24 in the Appendix) accounts for imperfect detection and reporting of new cases. Only symptomatic cases are assumed to be detectable in surveillance testing. Labels indicate the parameters and variables that affect the rates of the processes represented by the arrows. Red indicates the mixing population; yellow indicates the protected population; green indicates the quarantined population; white indicates the recovered population; black indicates the deceased population.

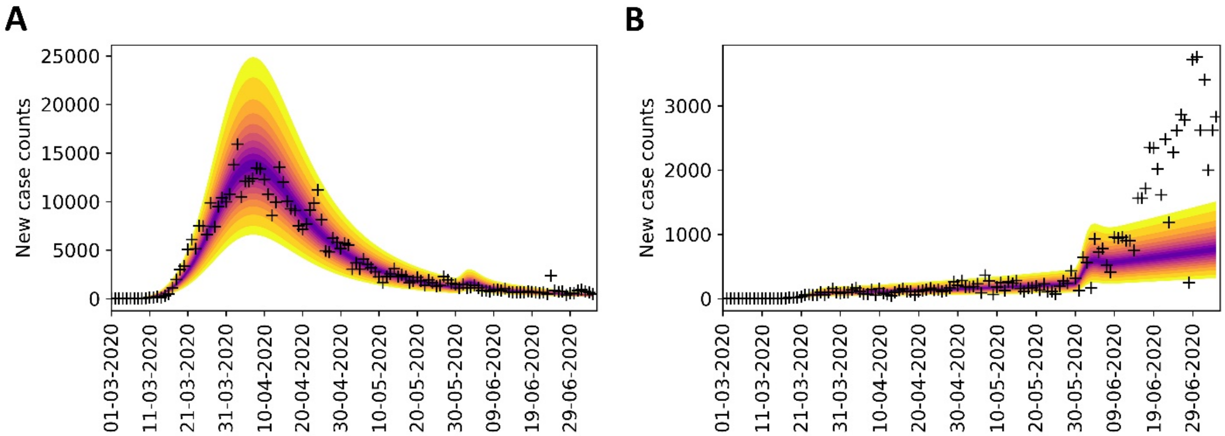

Appendix Figure 2. Potential transmission effects of a theoretical mass gathering on May 30, 2020 during regional coronavirus disease epidemics in the (A) New York City, NY and (B) Phoenix, AZ metropolitan statistical areas. Color bands indicate credible intervals for predictions of daily case reports. Crosses indicate observed data.

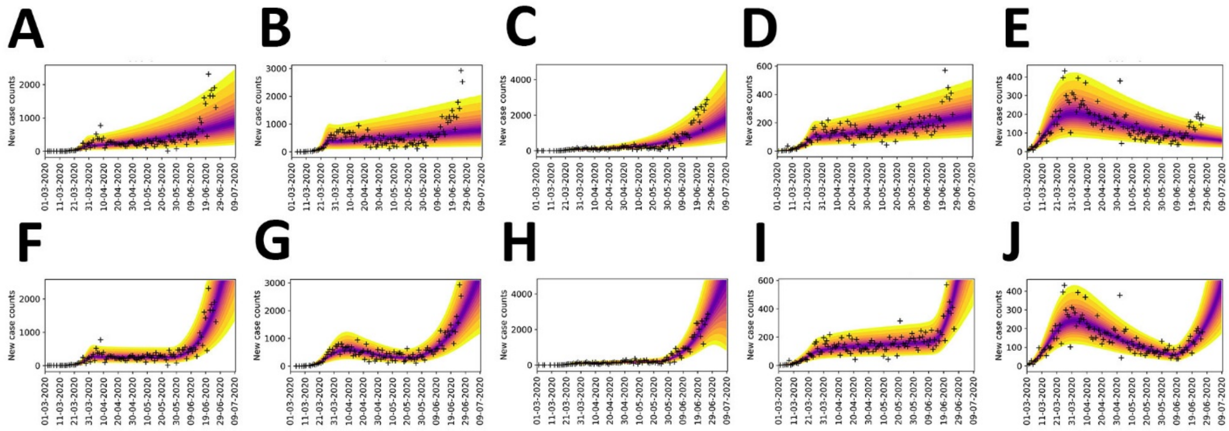

Appendix Figure 3. Predictive inferences conditioned on the compartmental model of daily new cases of coronavirus disease in 5 metropolitan statistical areas United States, 2020. A–E) Inferences of model with 1 initial social distancing period for A) Houston, TX; B) Miami, FL; C) Phoenix, AZ; D) San Francisco, CA; E) Seattle, WA. F–J) Inferences of model with 2 distinct social distancing periods for F) Houston, TX; G) Miami, FL; H) Phoenix, AZ; I) San Francisco, CA; J) Seattle, WA. Color bands indicate credible intervals for predictions of daily case reports. Crosses indicate observed data.

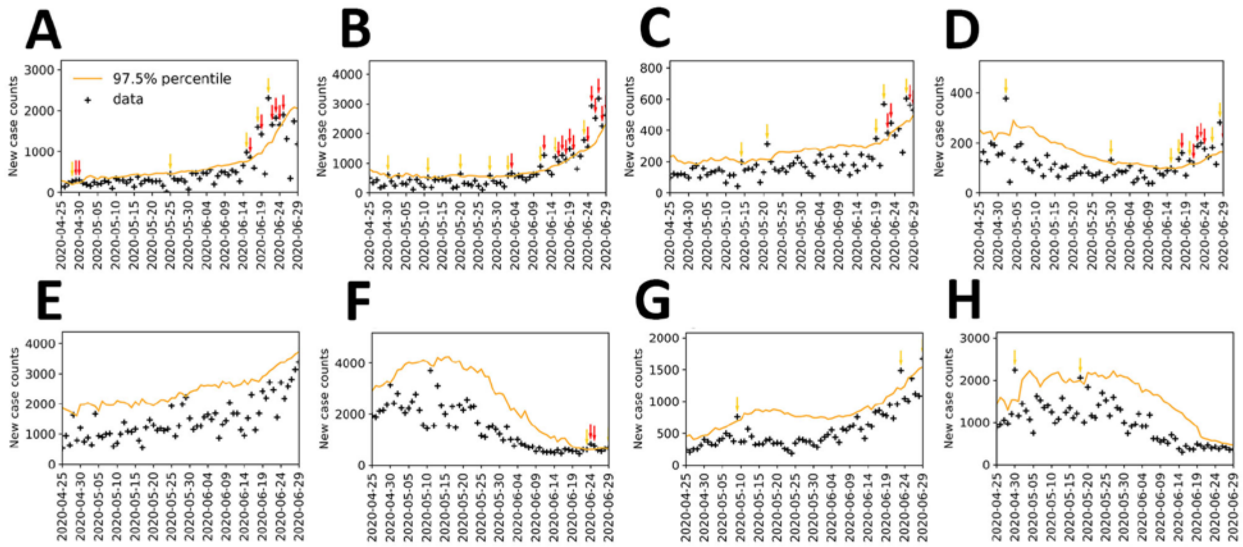

Appendix Figure 4. Comparison of next-day new case predictions and the corresponding empirical reported case counts of coronavirus disease in 5 metropolitan statistical areas, United States, 2020. A) Houston, TX; B) Miami, FL; C) San Francisco, CA; D) Seattle, WA; E) Los Angeles, CA; F) Chicago, IL; G) Dallas, TX; and H) Washington, DC. Crosses indicate observed daily case reports. Orange line indicates 97.5% probability percentile. Yellow arrows mark upward-trending rare events. Red arrows mark upward-trending anomalies.

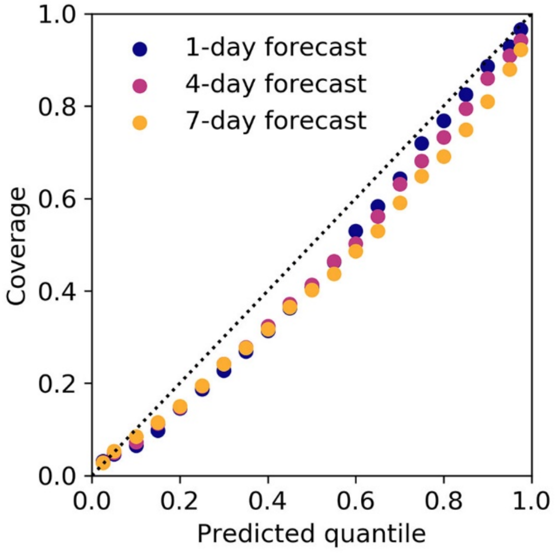

Appendix Figure 5. Out-of-sample validation of forecasting accuracy for region-specific compartmental models of daily new cases of coronavirus disease, United Sates, July 14–September 9, 2020. Empirical coverage was calculated for predicted detection of new cases 1, 4, and 7 days into the future. The dotted line indicates the coverage expected for unbiased prediction.

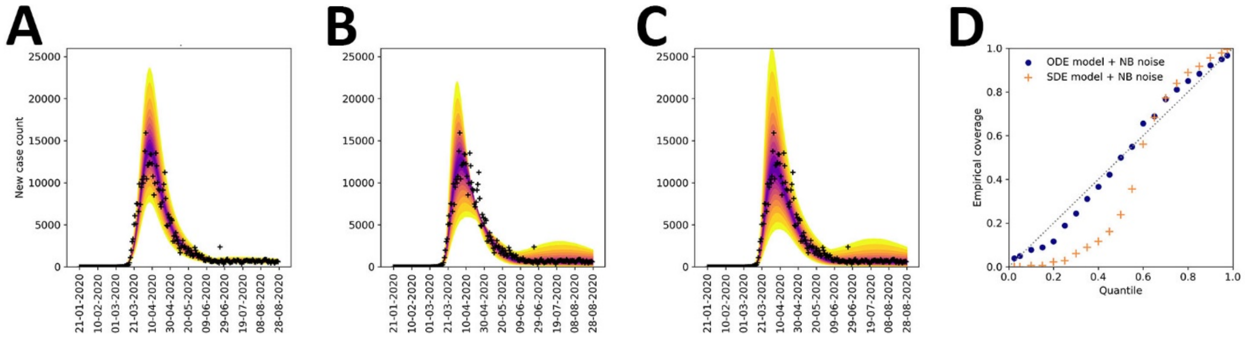

Appendix Figure 6. Comparison of forecasting accuracy of deterministic and stochastic versions of the compartmental model for daily new cases of coronavirus disease in the New York, NY metropolitan statistical area, United States, 2020. A) Results conditioned on the deterministic (i.e., ordinary differential equation) compartmental model. B, C) Results conditioned on a comparable stochastic differential equation model. D) In-sample validation results. Crosses indicate observed data. Colored bands indicate credible intervals of predictive posteriors. NB, observation noise; ODE, ordinary differential equation; SDE, stochastic differential equation.

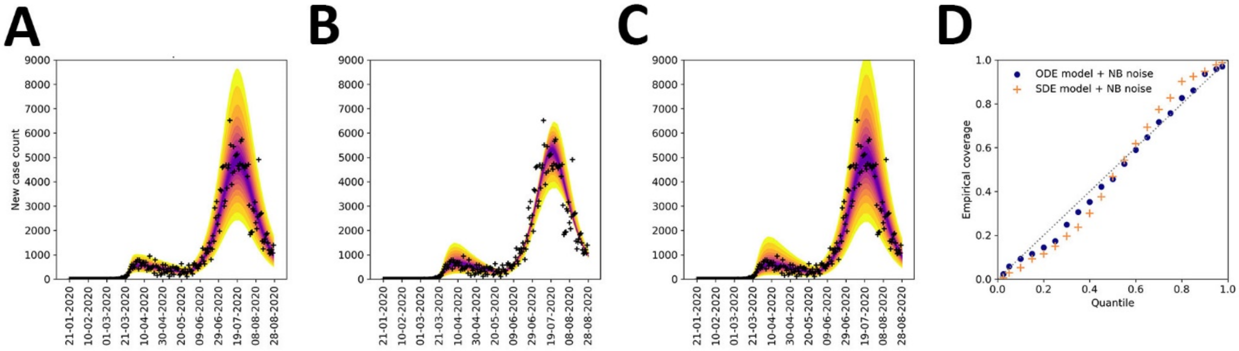

Appendix Figure 7. Comparison of forecasting accuracy of deterministic and stochastic versions of the compartmental model for daily new cases of coronavirus disease in the Miami, FL metropolitan statistical area, United States, 2020. A) Results conditioned on the deterministic (i.e., ordinary differential equation) compartmental model. B, C) Results conditioned on a comparable stochastic differential equation model. D) In-sample validation results. Crosses indicate observed data. Colored bands indicate credible intervals of predictive posteriors. NB, observation noise; ODE, ordinary differential equation; SDE, stochastic differential equation.

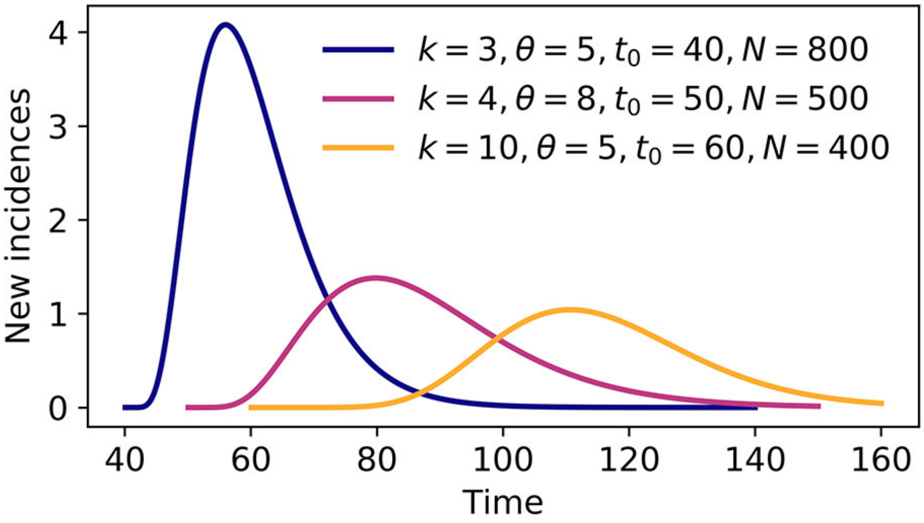

Appendix Figure 8. Illustration of shapes produced by fitting function to capture trends in regional coronavirus epidemic curves, United States, 2020.

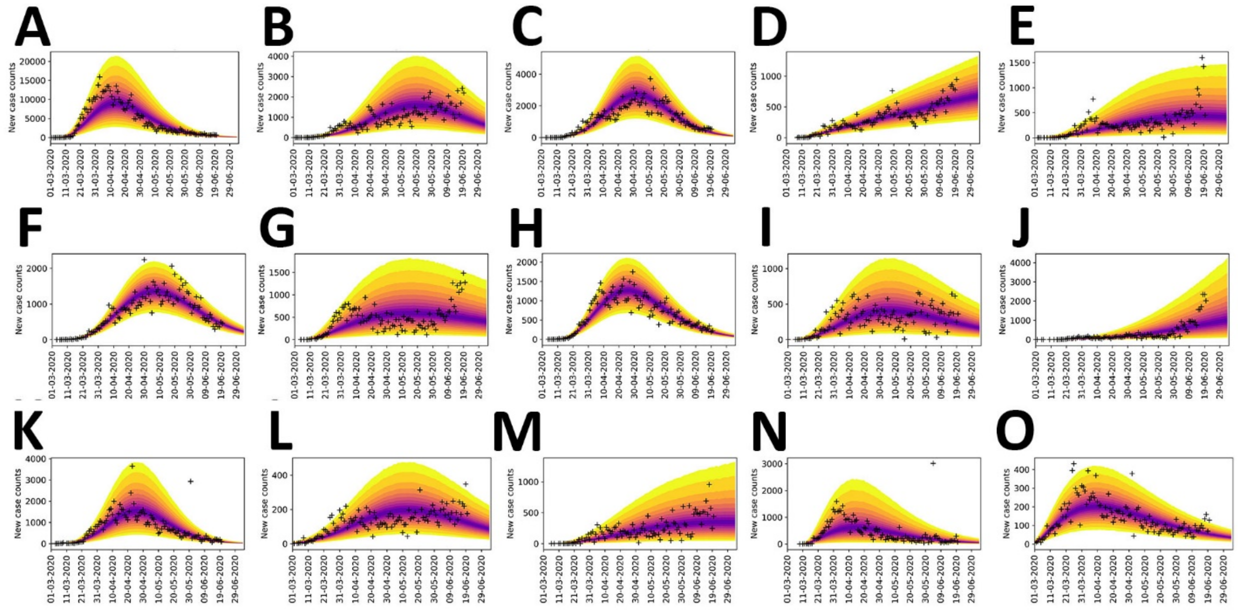

Appendix Figure 9. Bayesian predictive inferences for the 15 most populous metropolitan statistical areas, United States, 2020. Predictions conditioned on the curve-fitting model for A) New York City, NY; B) Los Angeles, CA; C) Chicago, IL; D) Dallas, TX; E) Houston, TX; F) Washington, DC; G) Miami, FL; H) Philadelphia; I) Atlanta, GA; J) Phoenix, AZ; K) Boston, MA; L) San Francisco, CA; M) Riverside, CA; N) Detroit, MI; and O) Seattle, WA. Color bands indicate credible intervals for predictions of daily case reports. Crosses indicate observed data.
