# Supplementary material for: Daily Forecasting of Regional Epidemics of Coronavirus Disease with Bayesian Uncertainty Quantification, United States
Source: Emerg Infect Dis. 2021 Mar;27(3):767–78. doi: 10.3201/eid2703.203364 (PMC7920670; doi:10.3201/eid2703.203364)
Supplement: Appendix 2 — Videos for daily forecasting of regional epidemics of coronavirus disease in the United States with Bayesian uncertainty quantification [file 20-3364-Techapp-s2.pdf]

Article DOI: https://doi.org/10.3201/eid2703.203364

Daily Forecasting of Regional Epidemics of Coronavirus Disease with Bayesian Uncertainty Quantification, United States

Appendix

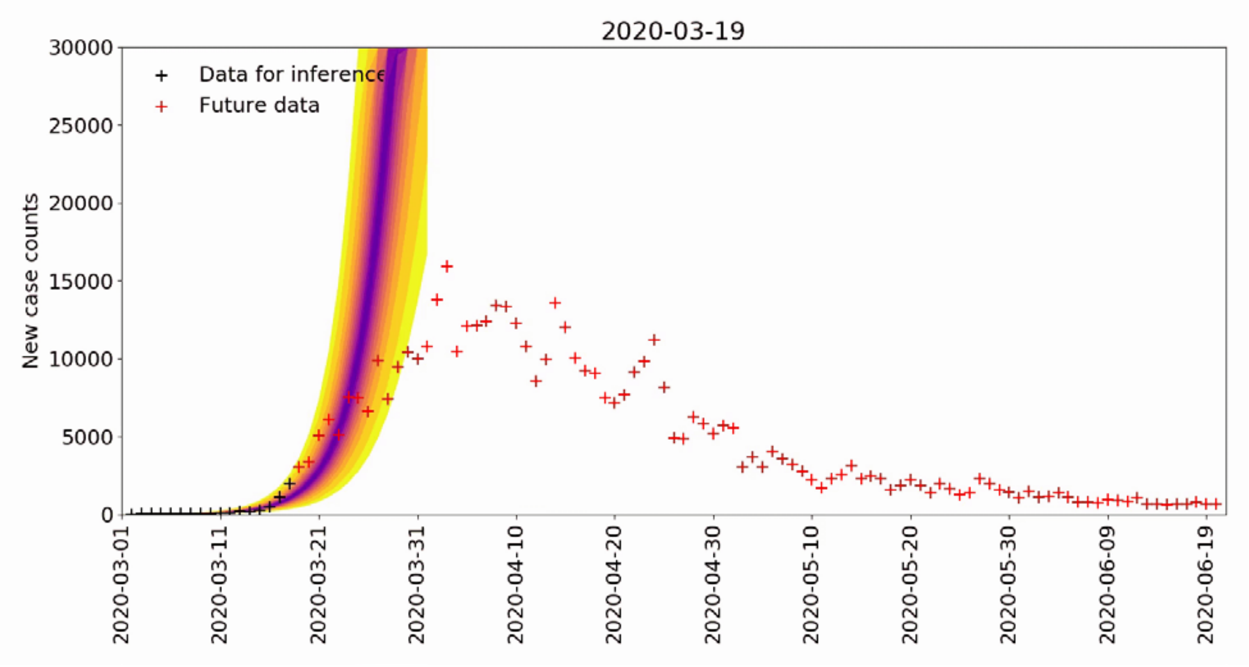

Appendix Video 1. Daily predictive inferences for new case counts of coronavirus disease in the metropolitan statistical area around New York City, New York, USA, March 19–June 6, 2020. Inferences are conditioned on the single-phase (*n* = 0) compartmental model. (Video, https://wwwnc.cdc.gov/eid/images/20-3364-App-video-1.gif)

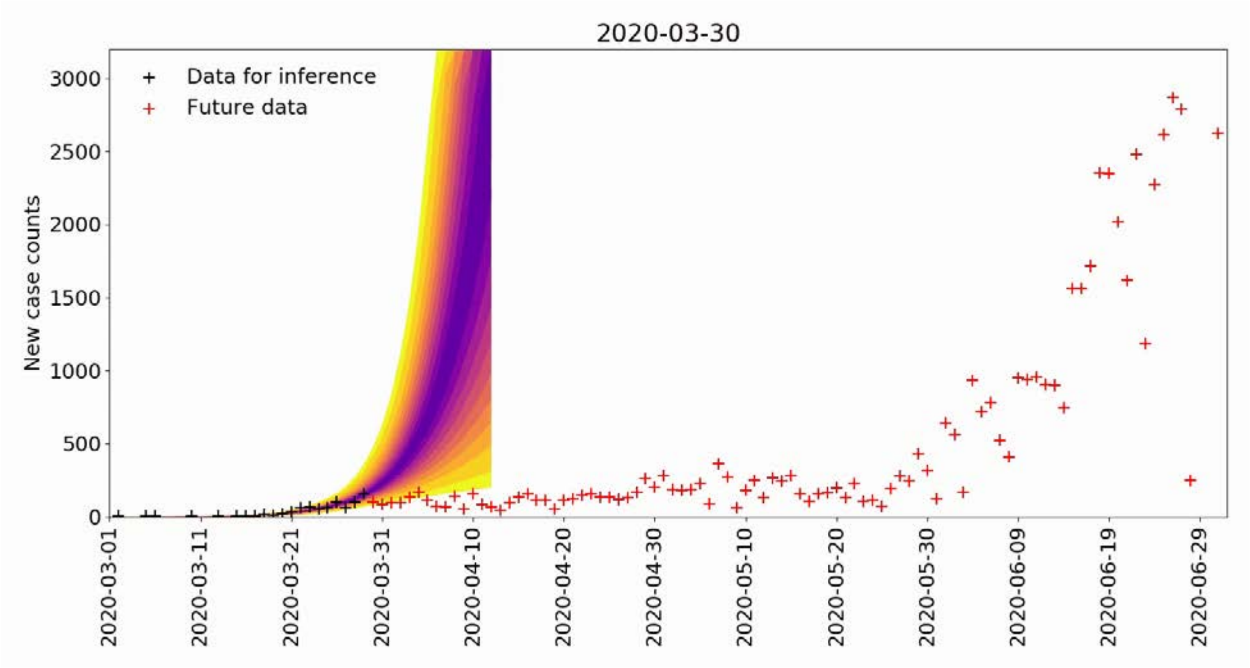

Appendix Video 2. Daily predictive inferences for new case counts of coronavirus disease in the metropolitan statistical area around Phoenix, Arizona, USA, March 30–June 17, 2020. Inferences are conditioned on the single-phase (*n* = 0) compartmental model. (Video, https://wwwnc.cdc.gov/eid/images/20-3364-App-video-2.gif)

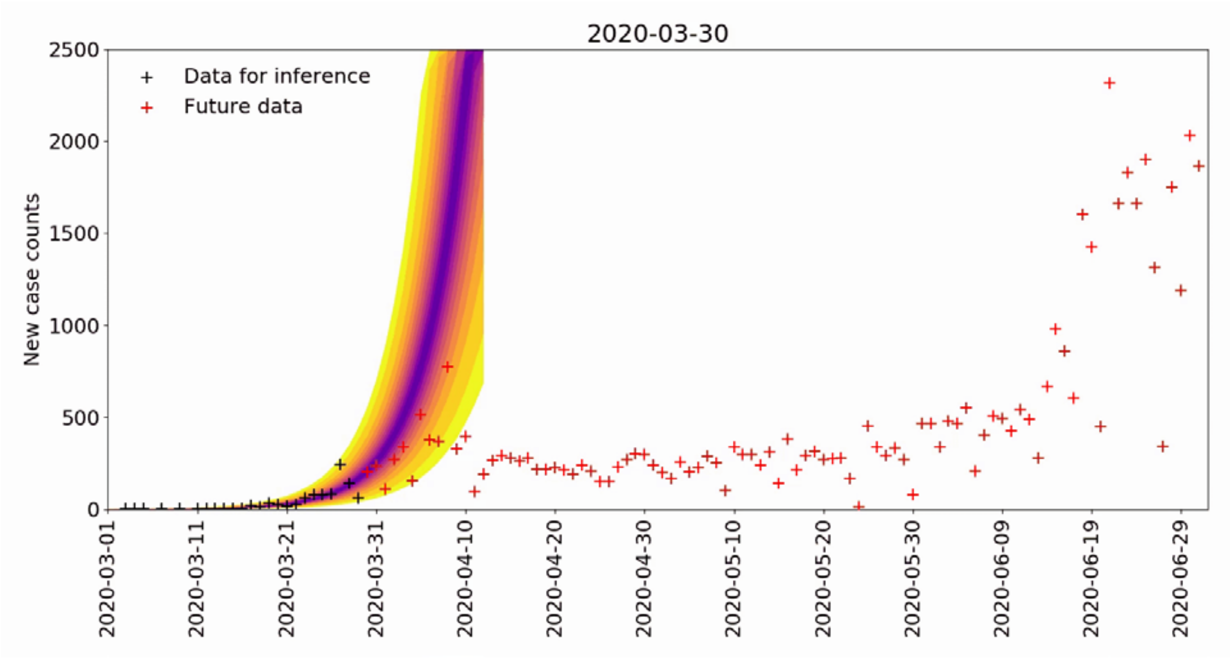

Appendix Video 3. Daily predictive inferences for new case counts of coronavirus disease in the metropolitan statistical area around Houston, Texas, USA, March 30–June 17, 2020. Inferences are conditioned on the single-phase (*n* = 0) compartmental model. (Video, https://wwwnc.cdc.gov/eid/images/20-3364-App-video-3.gif)

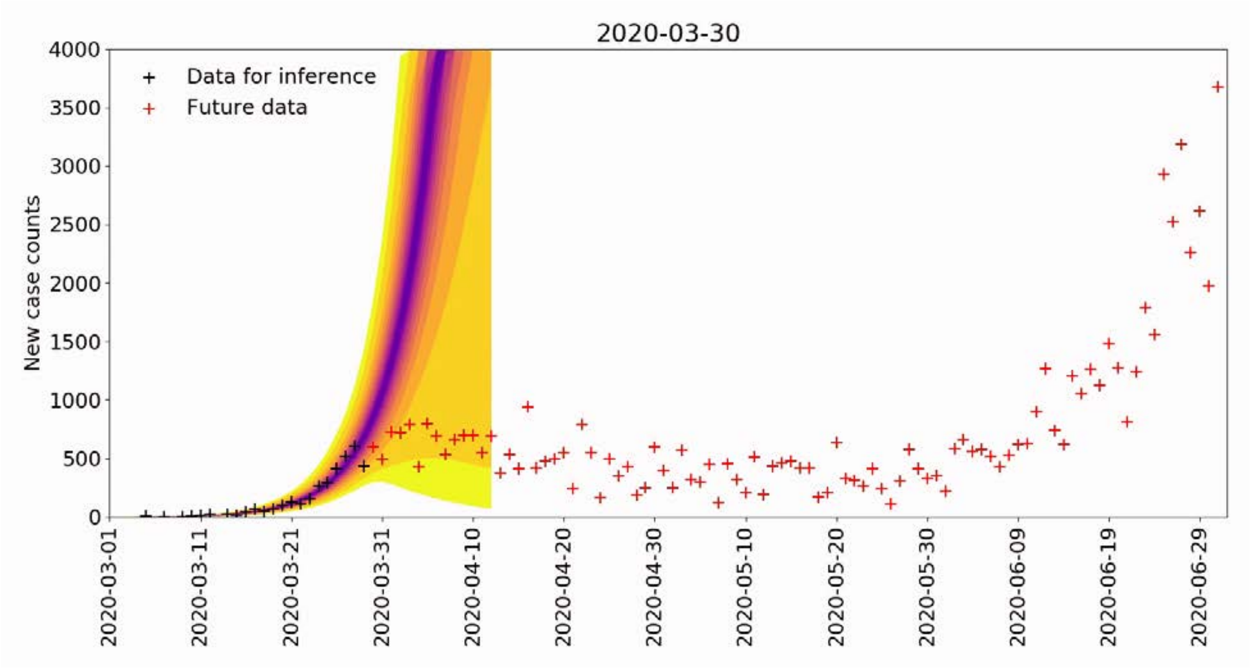

Appendix Video 4. Daily predictive inferences for new case counts of coronavirus disease in the metropolitan statistical area around Miami, Florida, USA, March 30–June 17, 2020. Inferences are conditioned on the single-phase (*n* = 0) compartmental model. (Video, https://wwwnc.cdc.gov/eid/images/20-3364-App-video-4.gif)

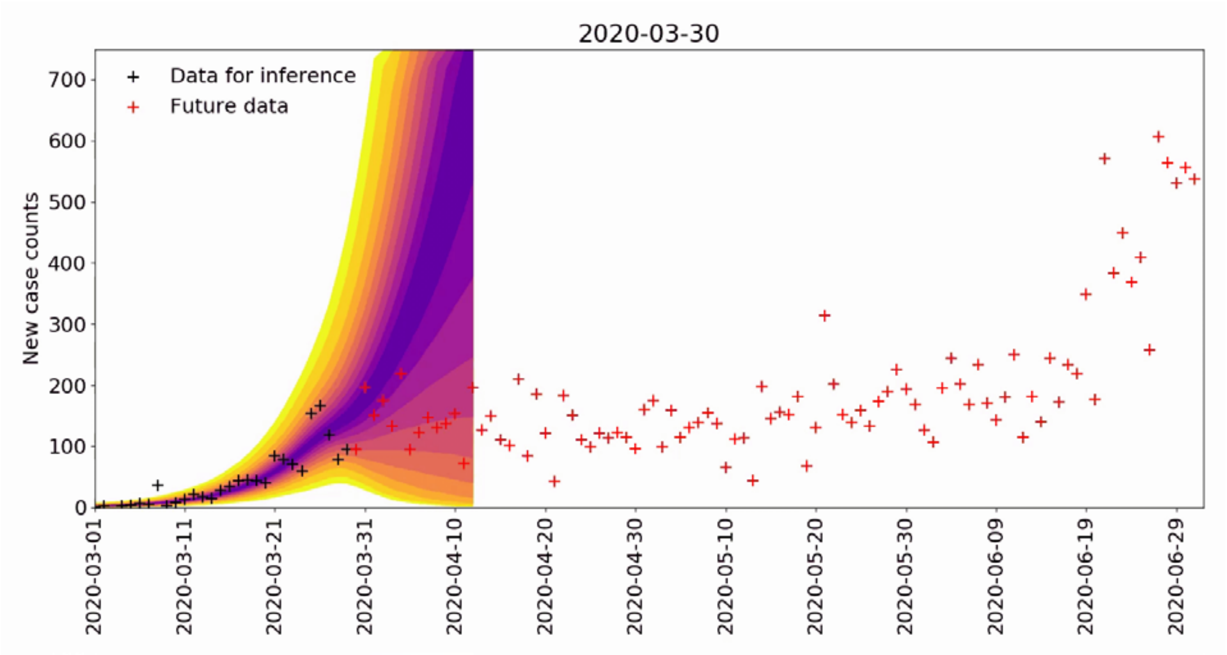

Appendix Video 5. Daily predictive inferences for new case counts of coronavirus disease in the metropolitan statistical area around San Francisco, California, USA, March 30–June 17, 2020. Inferences are conditioned on the single-phase (*n* = 0) compartmental model. (Video, https://wwwnc.cdc.gov/eid/images/20-3364-App-video-5.gif)

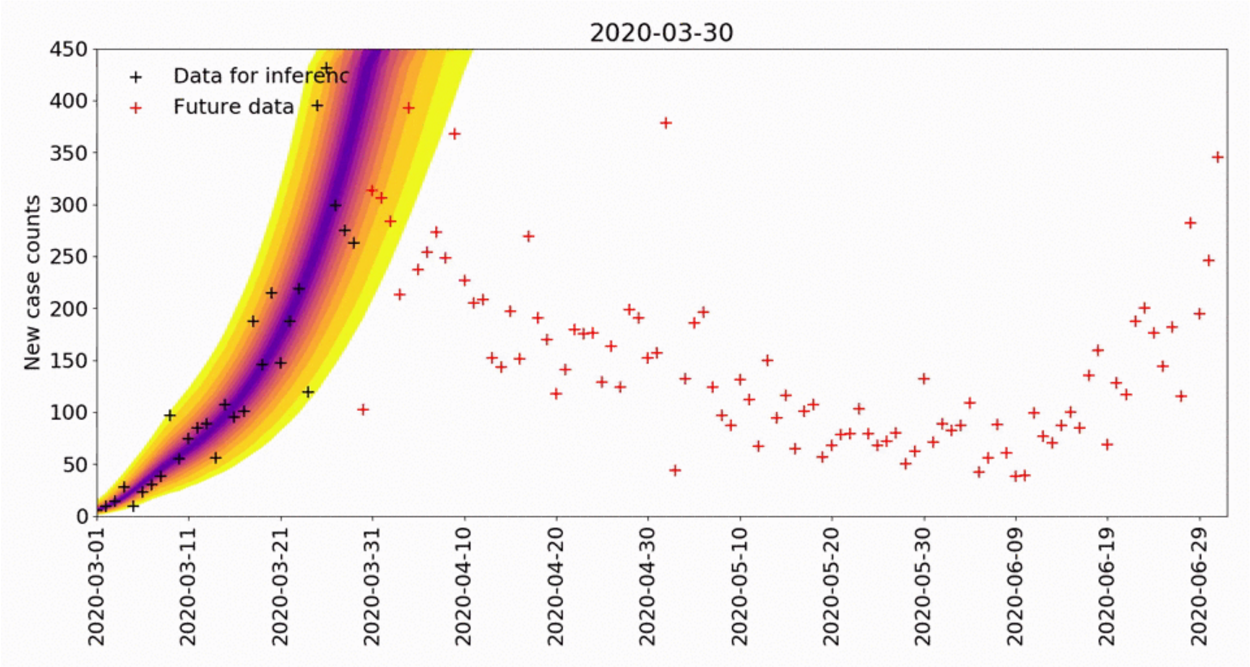

Appendix Video 6. Daily predictive inferences for new case counts of coronavirus disease in the metropolitan statistical area around Seattle, Washington, USA, March 30–June 17, 2020. Inferences are conditioned on the single-phase (*n* = 0) compartmental model. (Video, https://wwwnc.cdc.gov/eid/images/20-3364-App-video-6.gif)

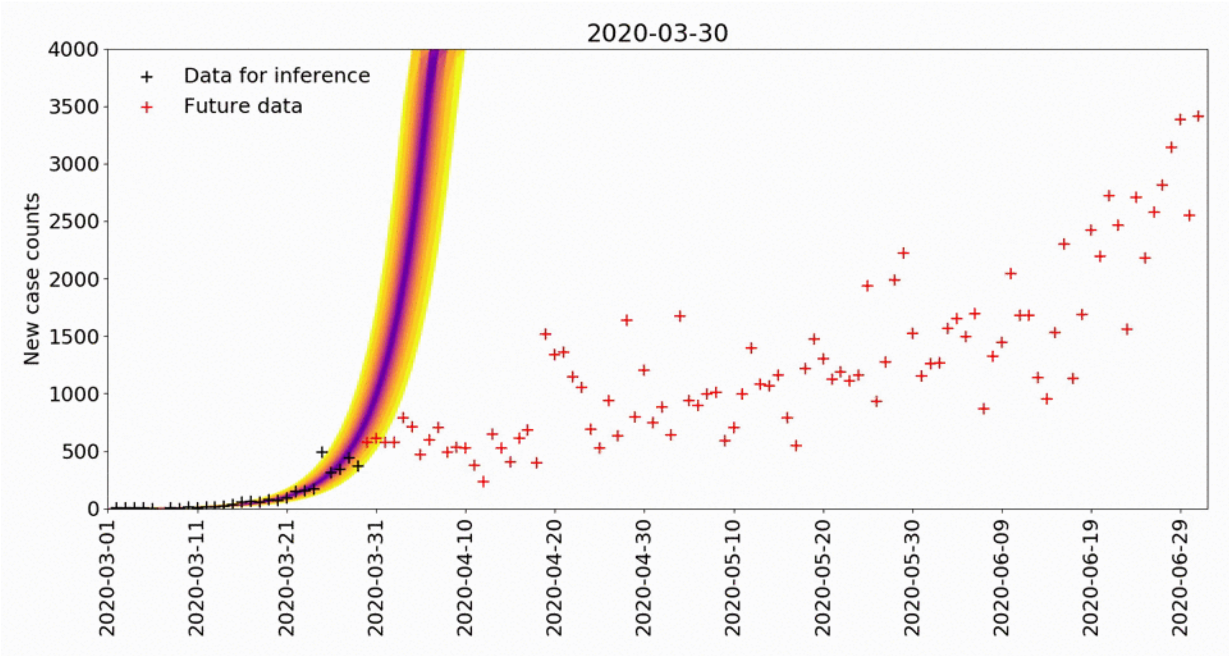

Appendix Video 7. Daily predictive inferences for new case counts of coronavirus disease in the metropolitan statistical area around Los Angeles, California, USA, March 30–June 17, 2020. Inferences are conditioned on the single-phase (*n* = 0) compartmental model. (Video, https://wwwnc.cdc.gov/eid/images/20-3364-App-video-7.gif)

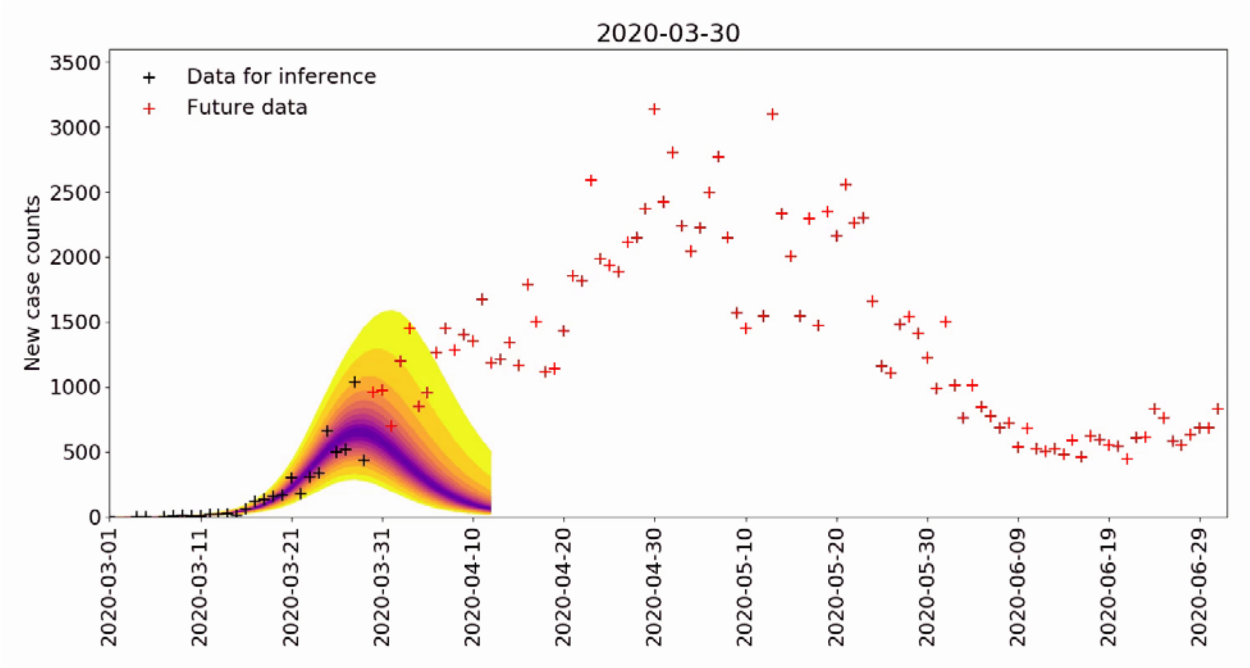

Appendix Video 8. Daily predictive inferences for new case counts of coronavirus disease in the metropolitan statistical area around Chicago, Illinois, USA, March 30–June 17, 2020. Inferences are conditioned on the single-phase (*n* = 0) compartmental model. (Video, https://wwwnc.cdc.gov/eid/images/20-3364-App-video-8.gif)

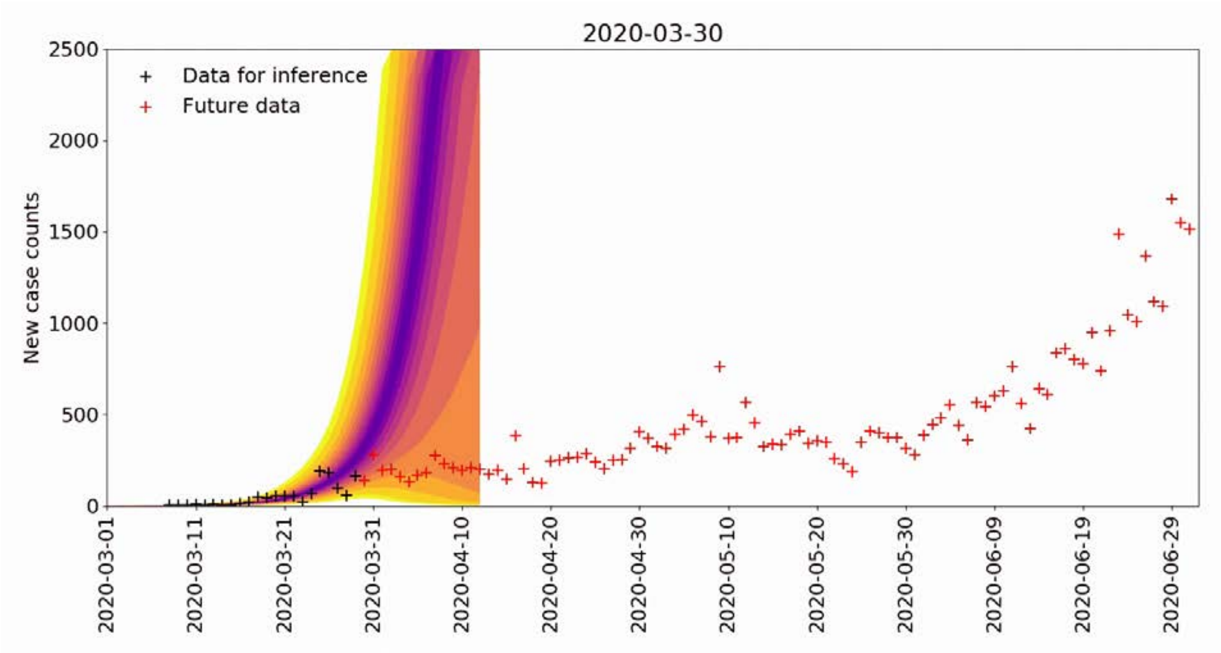

Appendix Video 9. Daily predictive inferences for new case counts of coronavirus disease in the metropolitan statistical area around Dallas, Texas, USA, March 30–June 17, 2020. Inferences are conditioned on the single-phase (*n* = 0) compartmental model. (Video, https://wwwnc.cdc.gov/eid/images/20-3364-App-video-9.gif)

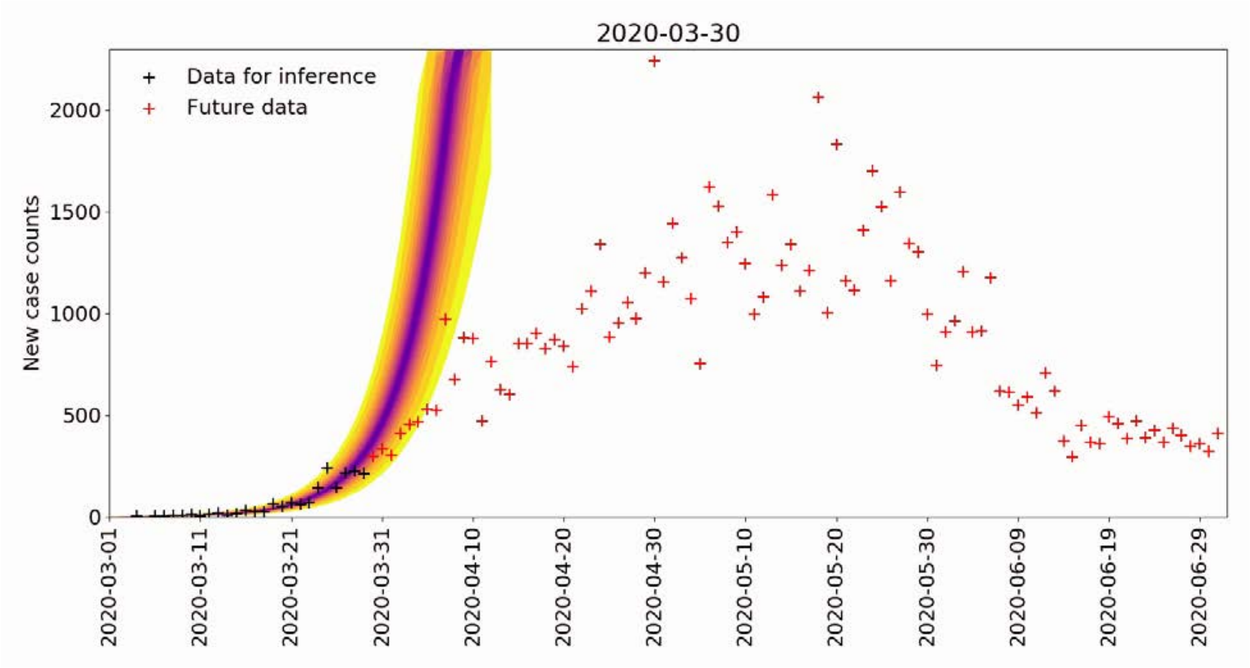

Appendix Video 10. Daily predictive inferences for new case counts of coronavirus disease in the metropolitan statistical area around Washington, DC, USA, March 30–June 17, 2020. Inferences are conditioned on the single-phase (*n* = 0) compartmental model. (Video, https://wwwnc.cdc.gov/eid/images/20-3364-App-video-10.gif)
